# Full Evaporative Vacuum Extraction—A Quantitative and Green Approach for Analysis of Semivolatile Organic Compounds in Drinking Water and Surface Water Using GC–MS

**DOI:** 10.1021/acs.analchem.2c03414

**Published:** 2023-02-07

**Authors:** Weier Hao, Daniel B. Cardin

**Affiliations:** Entech Instruments Inc, 2207 Agate Ct, Simi Valley, California, 93065, United States

## Abstract

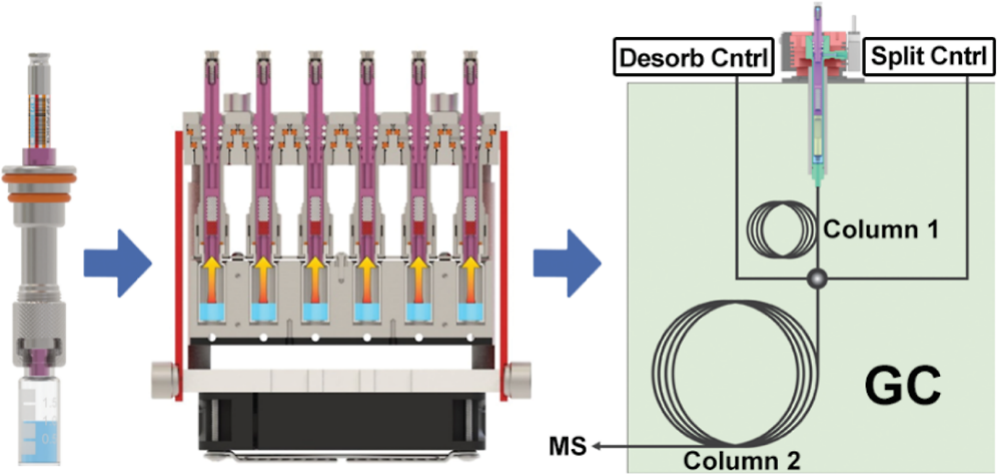

Full evaporative vacuum extraction (FEVE) was developed in this work for analysis of a broad range of semivolatile organic compounds (SVOCs) in drinking water and surface water. Sorbent pens are used in a two-stage process that first evaporates the sample matrix through sorbent beds under vacuum to recover the lighter SVOCs, followed by the application of a higher temperature and stronger vacuum to the sample vial to recover the remaining heavier SVOCs once the matrix has evaporated. After extraction, the sorbent pens are desorbed into a GC–MS using a uniquely designed “splitless” delivery system to maximize sensitivity. Critical extraction and desorption parameters that affect the method performance were optimized. After FEVE, the sorbent pens can be stored for 7–10 days at room temperature while maintaining a less than 15% loss in analyte recovery. As a proof of concept, 10 drinking water and surface water samples were analyzed using this method. 69 analytes were detected in these water samples, with the highest concentration of 1986 ng/L for bromacil. Heptachlor epoxide, chlorpyrifos, metolachlor, butachlor, and 2,3′,4′,5-tetrachlorobiphenyl were detected in four samples. None of the analytes were above the health and safety thresholds set by California Proposition 65.

## Introduction

For decades, extractions of semivolatile organic compounds (SVOCs) in aqueous matrices have been performed by liquid–liquid extraction and solid-phase extraction (SPE). These conventional extraction techniques have been applied in analytical laboratories worldwide and proven to be effective for routine water analysis. Nonetheless, with the development of new extraction techniques such as solid-phase micro-extraction (SPME) and stir-bar sorptive extraction (SBSE) in the 1990s, these conventional extraction methods have been critically challenged in sensitivity, efficiency, and environmental friendliness.^1,2^

SPME was first introduced in 1990 to address the growing need for rapid and solvent-free sample preparation.^1^ This technique provides simultaneous separation and preconcentration for volatile analytes in complex sample matrices. It has been considered an advanced technique over SPE due to generally shorter analysis time, simpler operation, and compatibility with automation. SPME’s green features such as reusable devices with an immobilized sorbent phase and the reduced generation of chemical wastes have also been welcomed by laboratories. However, this technique has limitations such as fragility of the needle and fiber, low chemical and temperature resistance, low extraction capacity, and relatively poor recoveries for compounds with low volatility.^3−7^

SBSE, first introduced in 1999, is a polymer-coated stir-bar technique that was designed to address some of the shortcomings of SPME.^2^ It offers advantages such as low detection limits, high recoveries for low-volatility compounds, and improved robustness.^2,8−13^ Nevertheless, this technique also has limitations. For example, it is generally not effective for extraction of relatively polar compounds due to the non-polar nature of polydimethylsiloxane (PDMS) coating, although alterations to the coating or samples matrix can be performed to increase recovery of certain polar compounds.^14^ SBSE recoveries are also subject to matrix effects, especially for samples with high organic matter, where adsorption of the analytes onto the organic matter can compete with the stir bars during the extraction.^15^ Furthermore, operations like removing the stir bars from the sample vial, rinsing, and drying are usually performed manually, which is laborious and can introduce errors.^9^ In addition, a multiple-step solvent soaking and high-temperature heating are required for clean-up of the stir bars.^16^

Vacuum-assisted sorbent extraction (VASE), a sorbent-based extraction technique recently developed, has been applied in various matrices as an alternative approach to SPME and SBSE.^17−19^ VASE utilizes sorbent-containing devices called Sorbent Pens (Entech Instruments, Simi Valley, CA) to perform headspace extraction under a vacuum condition. Each sorbent pen is packed with 100 mg of sorbent materials, which has a surface area approximately 10,000 times that of an SPME fiber.^17^ To accelerate the extraction kinetics, reduce the sampling time, and extend the range of analytes, the in-vial extraction is performed in a vacuum environment.^17^ After extraction, the sorbent pens are thermally desorbed using a specialized desorption system into a gas chromatography-mass spectrometer (GC-MS). Compared with SBSE and SPME, VASE has advantages such as higher durability, improved sensitivity due to larger sorbent surface area, reduced matrix interferences, and ability to use a series of sorbents in the sorbent pens to recover a wider range of compounds ranging from volatile to semivolatile and from polar to nonpolar.

In this work, full evaporative vacuum extraction (FEVE), an alternative to VASE, has been developed to speed up the extraction process and maximize method sensitivity specifically for samples containing low-suspended solids in a primarily volatile matrix such as water. FEVE employs similar sorbent devices to those used in VASE, but rather than maintaining a closed system during extraction, FEVE volatilizes the entire matrix through the sorbent beds to a vacuum pump. During water evaporation, the more volatile analytes are trapped by a stronger sorbent that is positioned behind a weaker sorbent. Once the water is fully evaporated, heat is applied to the sample vial to transfer less-volatile analytes into the vapor phase for capture predominantly by the weaker sorbent of the sorbent pen. This combination of vacuum evaporation, secondary heating, and multi-bed sorbent design enables extraction and preconcentration of a wide range of SVOCs in a single analysis. Unlike VASE and other extraction techniques, FEVE completely removes the liquid matrix in the sample and then heats the vial under vacuum to recover compounds that exhibit low volatility or high affinity to the sample matrix. As the matrix phase is eliminated from the system, the sorbents do not need to compete with the sample matrix for the analytes, thus reducing the matrix effect and enabling high recovery of a broad range of SVOCs. After extraction, the sorbent pens are sequenced for automated thermal desorption (TD) into a GC–MS for analysis.

US Environmental Protection Agency (EPA) Method 525 determines levels of SVOCs in drinking water, including polycyclic aromatic hydrocarbons (PAHs), organochlorine pesticides (OCPs), organophosphate pesticides (OPPs), organosulfur pesticides (OSPs), organonitrogen pesticides (ONPs), polychlorinated biphenyls (PCBs), phthalates, and others.^20^ These chemicals have been extensively applied, recognized as high-priority organic pollutants, and have raised serious environmental and human health concerns worldwide.^21−30^ An efficient and green method that can provide accurate, precise, sensitive, and quantitative measurements of these pollutants is needed. In this study, an FEVE–TD–GC–MS method was developed to analyze all of the more than 120 SVOCs listed by EPA Method 525 in drinking water and surface water. This list of analytes covers a wide range of SVOCs from lighter organophosphate chemicals such as diisopropyl methylphosphonate to heavier six-ring PAHs such as benzo[ghi]perylene.

## Experimental Section

### Design of FEVE

**Figure 1:**
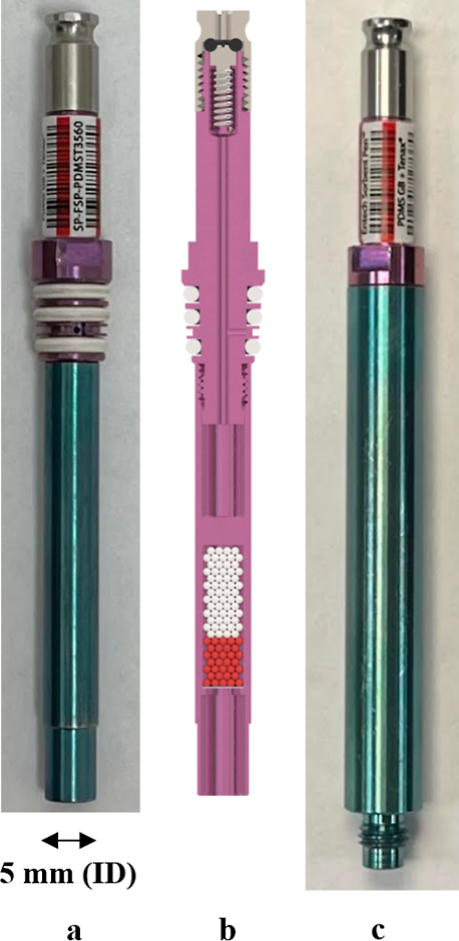
Photograph (a) and cross-section (b) of an FSP showing the internal sorbent beds: PDMS-coated glass beads (red) and Tenax (white). The FSPs are stored in a sleeve when not in use (c).

The FEVE sorbent pen (FSP) has a special design that extends into the neck of 2 mL FEVE sample vials to ensure recovery of heavy SVOCs. To capture a broad range of SVOCs, the sorbent bed consists of two sorbents in series: first, PDMS-coated glass beads and then 35/60 mesh Tenax TA. This FSP design is shown in Figure 1. PDMS-coated glass beads were chosen as a first bed to minimize the desorption heat needed to recover the heavier and the more thermally labile compounds, thereby optimizing their recoveries. For analysis of more volatile compounds, a stronger third sorbent like Carboxen or Carbosieve can be added in the FSP to capture these lighter compounds. However, for the suite of SVOCs in this study, a third sorbent was not necessary.

**Figure 2:**
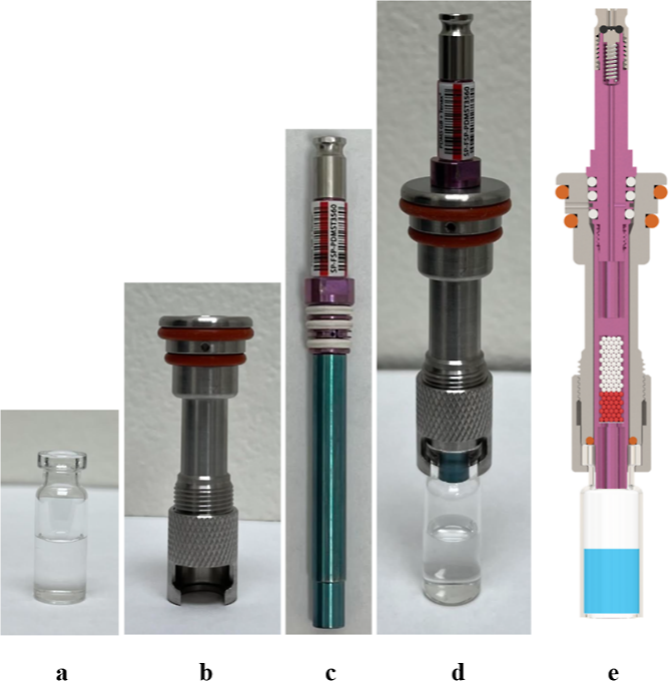
Components comprising the FEVE sample assembly, including 2 mL sample vial with 1 mL of the water sample (a), FEVE vacuum sleeve with a vial nut and silicone O-rings (b), and FSP (c). Photograph (d) and cross-section of the completed assembly showing the entrance of the FSP extending into the vial to ensure recovery of heavy SVOCs (e).

**Figure 3:**
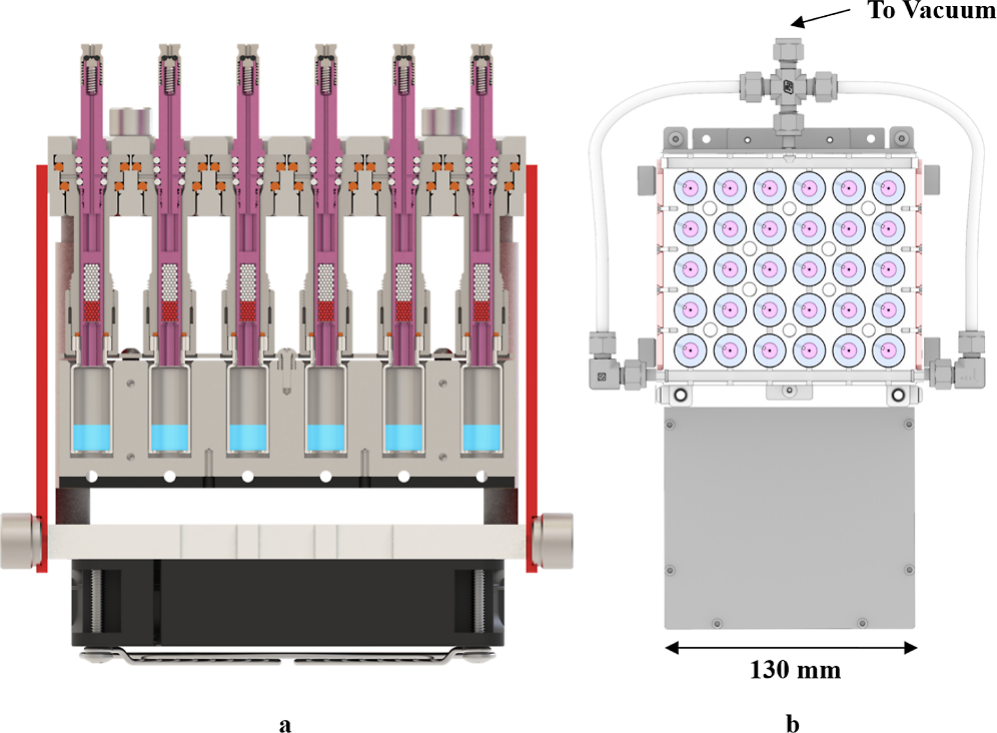
Front cross-section view of the FEVE module during extraction of multiple water samples (a). Top-down view of the FEVE top plate, FSP cooling fan, vacuum output, and 30 FEVE sample assemblies in place (b).

As shown in Figure 2, a 2 mL sample vial with 1 mL of the water sample was attached to an FEVE vacuum sleeve, and then, an FSP was inserted. A silicone O-ring was placed between the top of the sample vial and the bottom of the vacuum sleeve to create a leak-tight seal. The FEVE assemblies were placed into the extraction module shown in Figure 3. A top plate was used to compress the two upper vacuum sleeve O-rings against the vacuum manifold to ensure a leak-tight seal. The multi-position design of the manifold allows for up to 30 samples to be extracted simultaneously.

The FEVE process consists of four major steps: vacuum verification, matrix evaporation, high-vacuum dehydration, and high-temperature diffusive desorption. During vacuum verification, the FEVE instrument is pumped down through the low-vac valve to reach a target vacuum pressure. Then, the valve is turned off, and the rate of pressure increase is used to determine whether the system is leak-tight. After the vacuum verification standard is met, the matrix evaporation starts. The low-vac valve is left on to help slowly remove the water matrix under vacuum. When the pressure of the instrument drops below 10 Torr, the process advances to high-vacuum dehydration, where the high-vac valve is turned on to provide a stronger vacuum, pulling the remaining volatile matrix in the vial through the FSP sorbents to the pump. When the pressure drops to 1 Torr, the sample vials are heated at 200 °C for 7.5 min. This step helps transfer the heavier SVOCs from the vials to the sorbents. An FSP cooling fan is turned on at this stage to keep the FSP sorbent cool to maximize its adsorption capacity. After the vial heater is cooled down, the FSPs are ready for TD–GC–MS analysis. The entire FEVE process takes 4–6 h, depending on the number of samples extracted simultaneously. Extraction of 30 samples in 6 h equates to an average time of 12 min per sample. As this is approximately half the time of a standard GC cycle, one FEVE system can provide maximum throughput for two GC–MS systems.

### TD and GC–MS

After completion of the FEVE extraction process, the FSPs were loaded into a 30-position sorbent pen sample tray. The sample handling was performed using an SPR40 sample preparation rail (Entech Instruments) with full automation. The 5800-SPDU Sorbent Pen Desorption Unit (Entech Instruments) was used as the TD system to deliver the analytes to the GC–MS. A 7890B/5977C GC–MS (Agilent Technologies, Santa Clara, CA) was used for optimization of the extraction and desorption parameters. A Trace 1310/ISQ 7000 GC–MS (Thermo Fisher Scientific, Waltham, MA) with an advanced electron impact ion source operating in the selected ion monitoring mode was utilized to further optimize the detection limits of the method, evaluate the method, and analyze the water samples. A UAC-1MS precolumn (5 m × 0.53 mm × 0.15 μm, methylpolysiloxane; Quadrex Corp, Bethany, CT) was used to collect the SVOCs during sample desorption while using a 7–8 mL/min flow rate, with excess flow eliminated through a split tee positioned at the junction between the precolumn and the analytical column. The analytical column used for GC separation was an Agilent HP-5MS (30 m × 0.25 mm × 0.5 μm, 5%-phenyl-methylpolysiloxane). The carrier gas was helium, at a flow rate of 1.2 mL/min.

**Figure 4:**
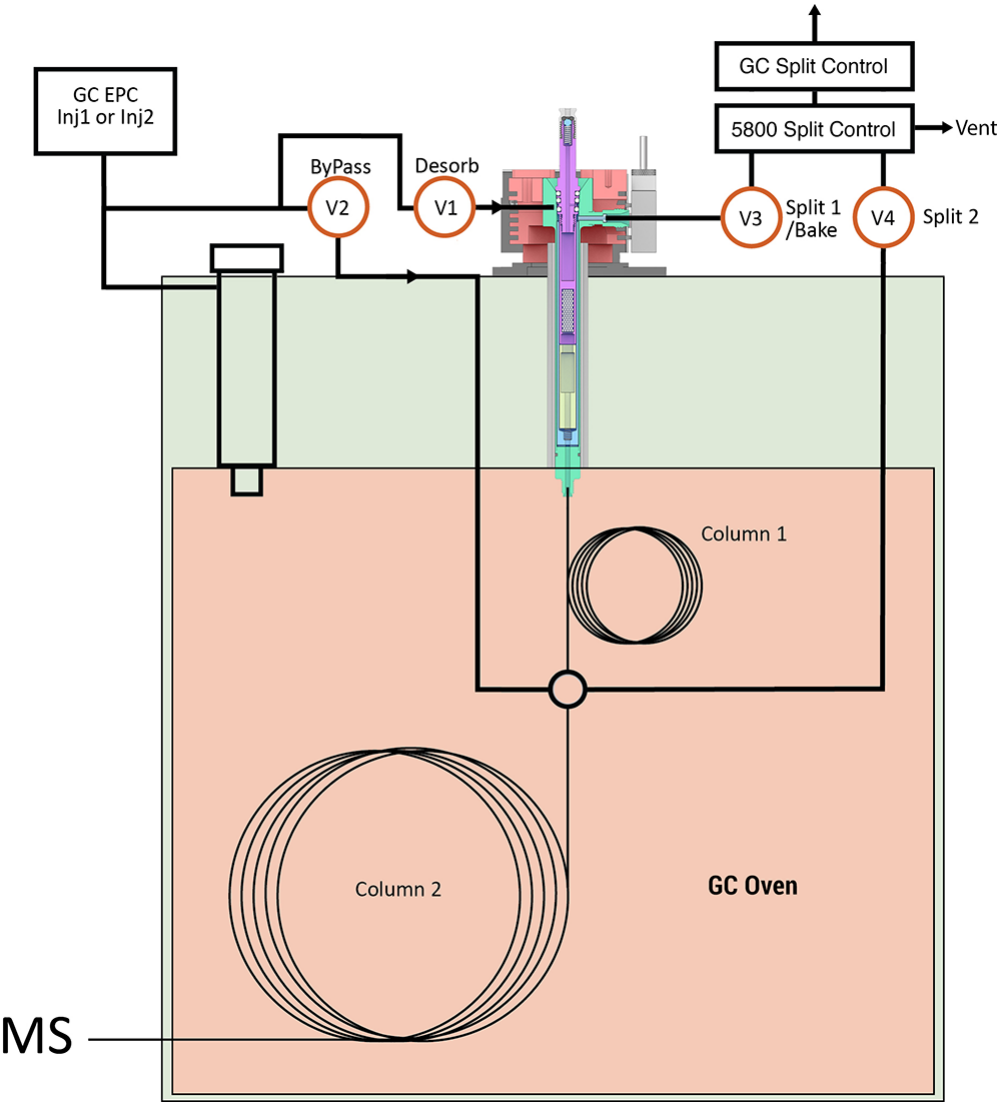
Configuration of the 5800-SPDU, two column design, and split control of the TD–GC–MS system. Column 1: Quadrex UAC-1MS (5 m × 0.53 mm × 0.15 μm). Column 2: Agilent HP-5MS (30 m × 0.25 mm × 0.5 μm).

Figure 4 shows the configuration of the 5800-SPDU, the precolumn (column 1), the analytical column (column 2), and the flow and split control. This design of the instrument enables sorbent pen pre-purging and pre-heating, desorption, GC delivery, split control, and residual backflushing during the analysis of each sample. After an FSP is inserted into the desorption unit, valves 2 and 4 are turned on during preheating, thereby bypassing the FSP. Once the desorption starts, valves 1 and 4 are turned on, enabling desorption flow through the FSP to deliver SVOCs to column 1. During desorption, compounds more volatile than the lightest analyte of interest are split out through valve 4. After desorption, valves 1 and 3 are tuned on, allowing the analytes to proceed splitlessly to column 2. By using a thicker film on column 2 than that on column 1, the analytes dynamically refocus on column 2, resulting in narrower chromatographic peaks. In practice, column 2 with a film thickness of 0.25 or 0.5 μm is recommended, while column 1 is 0.15 μm. The length of column 1 can be greater than 5 m if longer desorption time is needed. During transfer of the analytes in the two columns, the desorption unit is baked at 260 °C to eliminate potential carryover in the FSP. After the heaviest analyte of interest is eluted out from column 1 and starts separating on column 2, valves 2 and 3 are turned on to backflush unwanted heavy compounds out through the entrance of column 1. Finally, the desorption unit cools down and returns to the idle status where valves 2 and 4 are on and ready for the next sample. The valve controls and flow directions of each stage are shown in Figure S2 in the Supporting Information. The GC oven temperature was held at 40 °C for 3 min during sample desorption, ramped at 12 °C/min to 160 °C, then ramped at 8 °C/min to 320 °C, and held for 1 min until the end of the run. Data acquisition and analysis were performed using Agilent MassHunter Workstation, Thermo Chromeleon, Entech SPRINT software, and Microsoft Excel.

### Reagents and Chemicals

MS-grade acetone was obtained from Sigma-Aldrich (St. Louis, MO). Standards of the SVOC analytes and surrogates were obtained from AccuStandard (New Haven, CT). These standards were diluted to a concentration of 20 mg/L with acetone and stored in a freezer (−20 °C). Before analysis, these standards were further diluted with acetone to a 400 or 4 μg/L mix as working standards. The chemical information and the GC–MS parameters of these analytes are listed in Table S1 in the Supporting Information.

## Results and Discussion

### Optimization of Desorption Temperature

The desorption time was optimized to achieve maximum recovery for both the lighter and heavier target analytes. An ideal desorption period allows all the heavier compounds to be released from the FSPs and, in the meantime, prevents the lighter ones from reaching the end of the precolumn where they would split out through valve 4. When desorbed at 260 °C, 3 min of desorption time was found to provide optimal results. Therefore, the desorption time was set at 3 min when optimizing the desorption temperature. A 1 μL working standard mix was spiked into 1 mL of deionized water in a 2 mL glass sample vial. After extraction, the FSPs were desorbed at varied temperatures.

Desorption temperatures of 170, 200, 230, and 260 °C were used and compared for each SVOC category. The recoveries of these categories using different desorption temperatures are shown in Figure S3 in the Supporting Information. For OCPs, phthalates, and PCBs, the recoveries plateaued at 200 °C. These recoveries varied within 4% from 200 to 260 °C. For ONPs, OPPs, OSPs, and PAHs, the recoveries increased with rising desorption temperature from 170 to 260 °C, although the difference was not significant from 200 to 260 °C. At 260 °C, the recoveries of all categories reached a range of 91.4–106%. Another factor to consider was the degradation of Tenax, one of the packed sorbents in the FSPs. The breakdown products of Tenax generally do not affect the analysis of the analytes of interest. Nevertheless, a fast degradation may shorten the lifespan of the FSPs. It was found that the breakdown products of Tenax significantly increased when desorbed at a temperature higher than 260 °C. Therefore, a desorption temperature higher than 260 °C is not advised. With all these factors considered, 260 °C was selected as the optimal desorption temperature for all the analytes of interest.

### Selection of Sample Vials

Glass vials are known to contain silanol active sites on their surface, which can interact with certain analytes and lead to declined recoveries.^31−34^ As the FEVE completely removes the sample matrix for maximum recovery of the analytes, sample vial inertness can have substantial impacts on the recoveries. Five different brands of 2 mL sample vials were tested and compared. Brands A, B, C, and D are 11 mm crimp-top 2 mL glass vials from different manufacturers. Brand E is deactivated Silonite-coated glass vials developed by Entech Instruments. This treatment aims to cover free silanols on the inside wall surface of the glass vials and thus minimizes the potential interactions between the analytes and the silanol groups.

**Figure 5:**
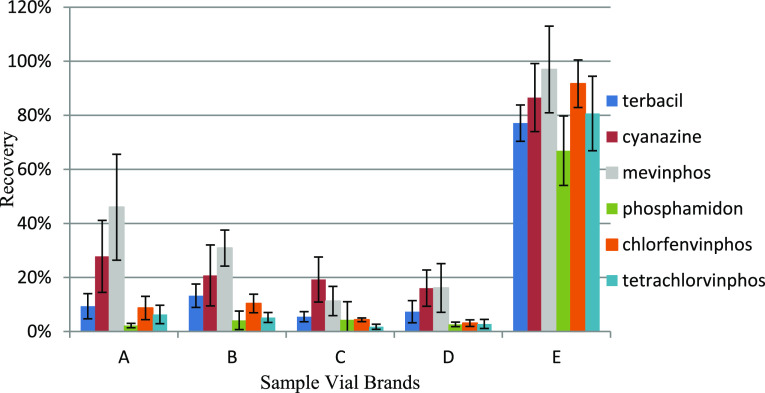
Recovery of terbacil, cyanazine, mevinphos, phosphamidon, chlorfenvinphos, and tetrachlorvinphos using five different brands of sample vials (*n* = 3, 95% CI).

Recoveries of PCBs, PAHs, and phthalates were not significantly affected by different vials. Nonetheless, for OCPs and OSPs, the mean recoveries using vials A–D were 82.6 and 82.1%, respectively, while using vial E were 98.2 and 94.3%, respectively. ONPs and OPPs appeared to be even more interactive with the silanol sites on the surface of the untreated glass vials. ONPs had a mean recovery of 67.3% with vials A–D, whereas it was improved to 92.6% with vial E. For OPPs, the recoveries using vials A–D were 45.7, 49.3, 39.9, and 48.7%, respectively. However, when using vial E, a 91.2% recovery was achieved. Several ONPs and OPPs had significantly low recoveries with vials A–D, namely, terbacil, cyanazine, mevinphos, phosphamidon, chlorfenvinphos, and tetrachlorvinphos. The recoveries of these compounds were on average 8.86, 21.0, 26.1, 3.33, 6.62, and 4.02%, respectively, using vials A–D. However, when using vial E, these recoveries increased to 77.1, 86.6, 97.0, 66.9, 91.7, and 80.7%, respectively. The recoveries of these analytes using vials A–E are shown in Figure 5.

### Optimization of FEVE High-Temperature Diffusive Desorption

**Figure 6:**
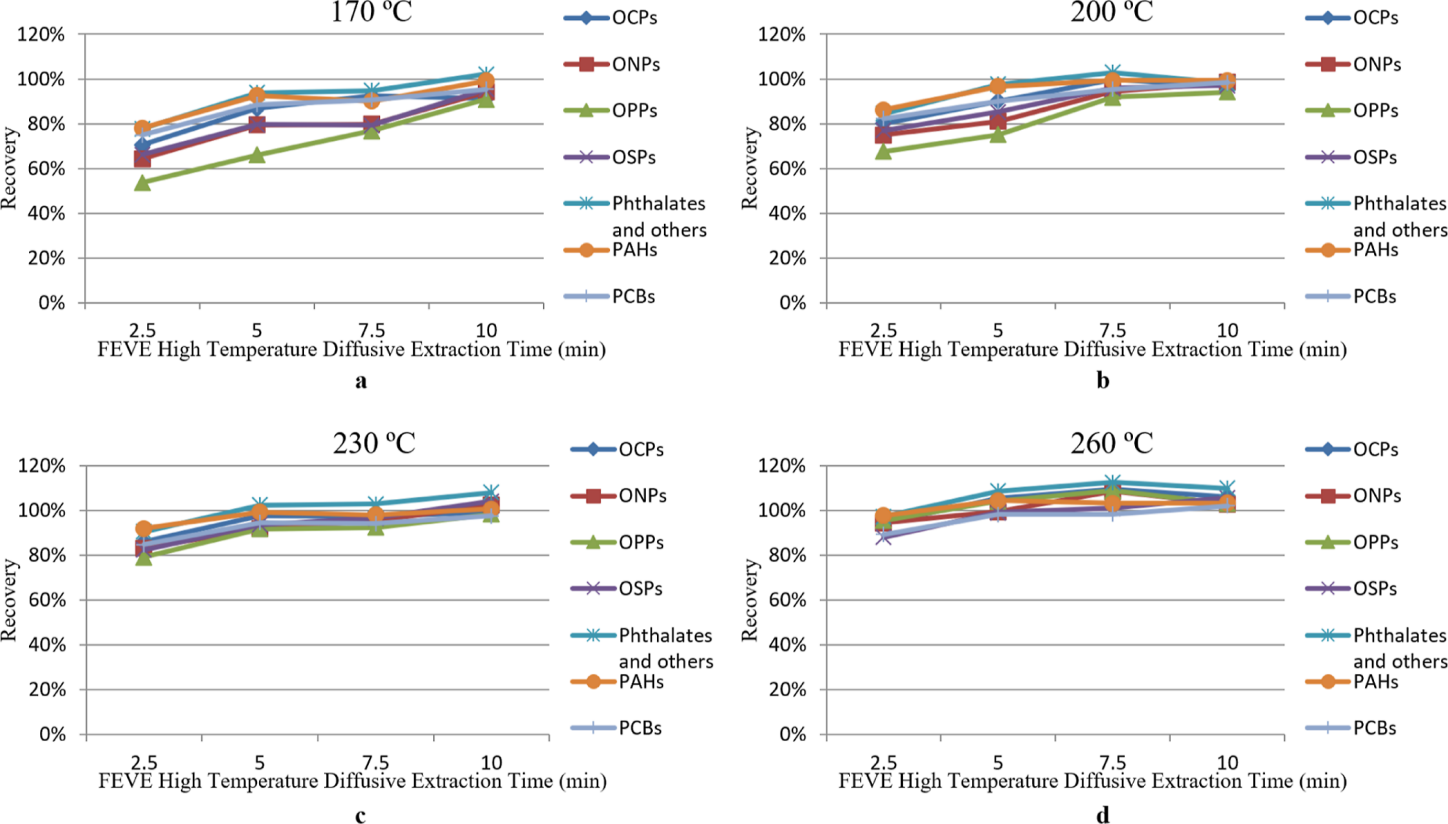
Recovery of OCPs, ONPs, OPPs, OSPs, phthalates and others, PAHs, and PCBs with different FEVE high-temperature diffusive desorption times at 170 (a), 200 (b), 230 (c), and 260 °C (d), *n* = 3.

The time and temperature of the FEVE high-temperature diffusive desorption are also essential parameters to optimize. Recoveries of OCPs, ONPs, OPPs, OSPs, phthalates, PAHs, and PCBs with different FEVE high-temperature diffusive desorption times and temperatures are shown in Figure 6. Extraction times of 2.5, 5, 7.5, and 10 min were investigated with temperatures of 170, 200, 230, and 260 °C. At 170 °C, for most categories, the recoveries kept increasing as the extraction time became longer. When extracted for 10 min, all categories had recoveries over 90%. At 200 °C, most categories reached maximum recoveries at 7.5 min. Among these categories, OPPs appeared to have stronger bonding interactions with the sample vials. When extracted at these lower temperatures, it took longer for these compounds to be released from the vials and captured by the sorbents. When extracted at 230 and 260 °C, similar trends were observed. The recoveries of all categories plateaued at 5 min, in a range of 91.9–105%. For all categories, no significant difference was observed when the high-temperature diffusive desorption was 200 °C, from 7.5 to 10 min or at 230–260 °C, from 5 to 10 min. However, a higher heating temperature can shorten the lifespan of the O-rings, create a higher level of siloxanes, and may cause breakdown of nano-plastic particles that have become ubiquitous. Thus, extracting for 7.5 min at 200 °C, an extraction condition with a relatively shorter time and lower temperature was selected.

### Method Performance Evaluation

Table 1 shows the limits of detection (LODs), calibration curve information, recoveries, and relative standard deviations (RSDs) of 123 target analytes listed in EPA Method 525. The method showed good linearity (*r*^2^ > 0.9900 for all analytes) and high sensitivity. LODs of most target analytes were in a range of 0.3–20 ng/L. Compared with those of SPME and SBSE methods using GC–quadrupole MS (QMS),^35−50^ these LODs were approximately 1–2 orders of magnitude lower. Analytes such as diethyl phthalate and dibutyl phthalate had higher LODs due to their high signal response in the blanks. Improved cleaning of O-rings and vacuum sleeves are being investigated to reduce the background levels. Analytes like phosphamidon and profenofos also had higher LODs due to relatively low response compared with those of the other compounds. Approaches such as further deactivation of the sample vials and the bottom of the FSPs and further removal of the moisture before heating during the FEVE process will be explored to further lower the LODs. GC–MS/MS systems capable of selected reaction monitoring can also be used to further investigate the minimum LOD levels achievable using the FEVE technique. Examples of the chromatography obtained for the analytes are shown in Figures S5 and S6 in the Supporting Information.

**Table 1:** Chemical Name, LODs, Calibration Curve Information, Recoveries, and RSDs of 123 Target SVOCs in Fortified Deionized Water Samples at Concentrations of 12, 60, and 300 ng/L (*n* = 7)

				12 ng/L		60 ng/L		300 ng/L	
	LOD (ng/L)	linear range (ng/L)	*r*^2^	recovery (%)	RSD (%)	recovery (%)	RSD (%)	recovery (%)	RSD (%)
DIMP	47.7	120–8000	0.9949	NA	NA	NA	NA	115	13.0
isophorone	18.2	40.0–8000	0.9982	NA	NA	107	5.07	103	8.21
dichlorvos	38.5	120–8000	0.9965	NA	NA	NA	NA	108	6.10
HCCPD	15.4	40.0–8000	0.9949	NA	NA	94.3	10.4	89.7	5.88
EPTC	11.0	40.0–8000	0.9960	NA	NA	113	17.6	109	12.2
mevinphos	20.6	40.08000	0.9990	NA	NA	81.7	28.7	78.0	25.7
butylate	12.9	40.0–8000	0.9941	NA	NA	106	7.19	102	2.66
vernolate	19.0	40.0–8000	0.9985	NA	NA	92.6	4.56	95.2	7.04
dimethyl phthalate	7.31	20.0–8000	0.9993	NA	NA	114	4.99	101	2.97
2,6-dinitrotoluene	11.0	20.0–8000	0.9931	NA	NA	118	14.9	108	11.9
etridiazole	13.6	20.0–8000	0.9985	NA	NA	92.7	15.2	94.6	12.1
pebulate	15.3	40.0–8000	0.9992	NA	NA	101	6.28	102	10.8
acenaphthylene	8.93	20.0–8000	0.9990	NA	NA	107	4.31	98.8	4.75
chloroneb	18.9	40.0–8000	0.9978	NA	NA	85.7	8.90	83.6	3.76
BHT	3.61	20.0–8000	0.9967	103	26.7	109	9.40	97.9	3.90
2-chlorobiphenyl	2.04	8.00–8000	0.9985	113	7.75	108	6.15	102	2.44
tebuthiuron	10.6	20.0–8000	0.9997	NA	NA	89.6	13.3	87.2	15.7
2,4-dinitrotoluene	12.9	40.0–8000	0.9910	NA	NA	123	26.8	102	16.6
molinate	1.19	8.00–8000	0.9973	NA	NA	105	16.2	106	13.0
DEET	16.8	40.0–8000	0.9996	NA	NA	100	13.6	94.6	7.50
diethyl phthalate	78.3	120–8000	0.9939	NA	NA	NA	NA	124	23.0
4-chlorobiphenyl	3.18	8.00–8000	0.9965	110	13.4	106	7.99	99.0	1.57
propachlor	20.4	20.0–8000	0.9973	NA	NA	105	12.6	100	8.05
fluorene	4.82	20.0–8000	0.9983	NA	NA	98.2	5.07	97.0	2.02
ethoprop	17.5	40.0–8000	0.9952	NA	NA	92.1	15.9	97.2	11.4
cycloate	12.4	40.0–8000	0.9973	NA	NA	97.0	8.77	99.8	3.18
chlorpropham	16.0	40.0–8000	0.9964	NA	NA	119	11.8	116	7.72
trifluralin	1.77	8.00–4000	0.9921	92.4	29.1	85.5	26.1	91.7	27.3
phorate	22.2	20.0–8000	0.9981	NA	NA	98.5	14.0	95.3	4.86
a-HCH	9.38	20.0–8000	0.9948	NA	NA	95.9	7.32	94.1	2.10
2,4′-dichlorobiphenyl	2.93	8.00–8000	0.9952	109	9.27	107	12.9	100	7.53
atraton	2.35	8.00–8000	0.9916	96.1	24.5	98.9	10.0	90.6	9.74
hexachlorobenzene	2.67	8.00–8000	0.9968	99.7	18.1	94.8	8.35	96.6	4.04
prometon	11.7	40.0–8000	0.9961	NA	NA	103	3.50	102	5.98
simazine	9.46	20.0–8000	0.9955	NA	NA	92.3	7.02	91.1	5.74
dimethipin	29.4	80.0–8000	0.9977	NA	NA	87.0	14.2	86.4	9.47
atrazine	3.67	8.00–8000	0.9932	106	12.2	98.7	10.0	97.8	14.9
propazine	1.55	8.00–8000	0.9962	99.1	9.90	100	8.79	97.0	10.1
b-HCH	13.4	20.0–8000	0.9976	NA	NA	96.4	8.15	90.5	6.25
pentachlorophenol	17.0	40.0–8000	0.9946	NA	NA	91.0	12.0	88.9	13.9
*d*-HCH	7.88	20.0–8000	0.9963	NA	NA	92.7	6.95	90.0	2.84
pronamide	15.0	40.0–8000	0.9971	NA	NA	95.9	9.6	94.5	9.6
chlorothalonil	17.8	40.0–8000	0.9911	NA	NA	83.5	22.2	86.9	4.28
2,2′,5-trichlorobiphenyl	1.16	8.00–8000	0.9983	104	17.1	102	8.63	96.7	7.13
terbacil	14.7	40.0–8000	0.9967	NA	NA	81.3	19.3	83.5	16.9
disulfoton	3.69	8.00–8000	0.9991	86.9	16.9	95.5	10.6	94.2	9.48
phenanthrene	1.50	8.00–8000	0.9995	113	7.17	108	7.44	102	3.29
g-HCH	8.12	20.0–8000	0.9975	NA	NA	93.1	8.81	91.4	7.93
anthracene	7.28	20.0–8000	0.9966	NA	NA	106	7.36	101	5.88
phosphamidon	91.6	120–8000	0.9921	NA	NA	NA	NA	63.3	33.5
acetochlor	2.83	8.00–8000	0.9956	91.5	26.3	87.3	13.5	91.2	44.5
vinclozolin	12.2	40.0–8000	0.9945	NA	NA	74.4	15.0	77.5	10.6
2,4,4′-trichlorobiphenyl	0.371	8.00–8000	0.9959	99.0	15.1	98.7	7.27	95.9	3.36
simetryn	3.51	8.00–8000	0.9935	104	9.48	98.6	4.58	101	8.72
alachlor	3.26	8.00–8000	0.9965	96.1	24.8	98.2	10.9	90.1	8.94
ametryn	9.94	20.0–8000	0.9955	NA	NA	102	7.72	97.3	13.5
parathion methyl	6.01	8.00–8000	0.9970	NA	NA	83.8	7.47	87.4	8.73
prometryne	1.98	8.00–8000	0.9990	104	11.0	99.8	8.47	99.7	9.77
heptachlor	0.993	8.00–8000	0.9972	102	19.8	100	12.1	97.7	13.6
bromacil	16.5	40.0–8000	0.9955	NA	NA	85.4	11.2	82.0	4.78
terbutryn	3.11	8.00–8000	0.9931	94.2	9.50	92.8	6.04	91.4	10.7
dibutyl phthalate	21.6	120–8000	0.9989	NA	NA	NA	NA	122	12.5
2,2′,5,5′-tetrachlorobiphenyl	0.757	8.00–8000	0.9961	92.6	10.0	95.0	6.21	93.5	3.17
cyanazine	16.3	40.0–8000	0.9966	NA	NA	84.6	19.5	81.7	12.6
chlorpyrifos	15.6	40.0–8000	0.9942	NA	NA	103	14.7	101	11.7
metolachlor	1.38	8.00–8000	0.9996	96.3	15.2	96.9	7.78	92.6	10.2
dacthal	0.309	8.00–8000	0.9947	101	12.2	96.6	6.57	93.2	7.07
triadimefon	18.2	40.0–4000	0.9975	NA	NA	98.6	9.25	101	9.81
2,2′,3,5′-tetrachlorobiphenyl	0.563	8.00–8000	0.9993	97.2	7.59	97.3	7.37	92.5	3.23
parathion	5.76	8.00–8000	0.9948	NA	NA	90.5	5.22	91.9	5.03
aldrin	3.58	8.00–8000	0.9972	92.3	21.3	96.5	9.85	92.3	5.36
diphenamid	7.05	20.0–8000	0.9958	NA	NA	98.1	7.67	96.1	2.66
MGK264(a)	12.5	20.0–8000	0.9946	NA	NA	85.1	13.1	84.4	11.0
MGK264(b)	13.9	20.0–8000	0.9970	NA	NA	82.5	9.48	79.9	9.96
chlorfenvinphos	16.3	40.0–8000	0.9920	NA	NA	74.8	12.7	76.6	14.5
heptachlor epoxide	1.87	8.00–8000	0.9934	91.0	17.6	84.3	9.07	86.0	11.8
2,3’,4′,5-tetrachlorobiphenyl	0.841	8.00–8000	0.9988	102	5.47	99.9	5.91	94.9	12.1
tetrachlorvinphos	11.1	20.0–8000	0.9980	NA	NA	82.8	25.0	81.1	22.0
trans-chlordane	0.885	8.00–8000	0.9922	105	6.95	98.9	9.99	96.7	10.0
butachlor	6.65	20.0–8000	0.9929	NA	NA	106	6.34	99.9	15.9
cis-chlordane	0.987	8.00–8000	0.9961	89.5	5.47	94.9	9.53	93.3	6.22
pyrene	2.83	8.00–8000	0.9990	112	4.28	116	9.62	94.9	5.59
profenofos	81.9	120–8000	0.9995	NA	NA	NA	NA	81.1	9.23
endosulfan I	17.8	40.0–8000	0.9975	NA	NA	86.4	10.5	87.1	7.76
napropamide	1.57	8.00–8000	0.9957	84.2	9.62	88.1	9.22	84.8	5.20
trans-nonachlor	1.97	8.00–8000	0.9936	103	5.70	96.0	8.99	94.2	5.14
tribufos	20.2	40.0–8000	0.9917	NA	NA	90.1	5.50	86.3	12.2
4,4′-DDE	1.98	8.00–8000	0.9995	100	2.52	96.6	7.61	96.1	5.36
2,3,3’,4′,6-pentachlorobiphenyl	0.603	8.00–8000	0.9952	94.2	7.34	97.8	7.48	99.9	5.00
dieldrin	13.5	40.0–8000	0.9987	NA	NA	102	7.04	107	6.14
nitrofen	22.2	40.0–8000	0.9951	NA	NA	76.6	16.7	80.9	8.86
oxyfluorfen	4.02	8.00–8000	0.9940	77.8	14.6	84.2	9.35	83.6	8.45
2,2’,3,4′,5′6-hexachlorobiphenyl	0.893	8.00–8000	0.9995	98.4	4.63	96.0	10.0	91.2	7.70
chlorobenzilate	12.2	20.0–8000	0.9990	NA	NA	87.7	10.7	90.0	8.23
endrin	3.21	8.00–8000	0.9936	81.0	17.9	85.7	13.8	80.7	14.1
2,3’,4,4′,5-pentachlorobiphenyl	0.532	8.00–8000	0.9950	94.8	3.60	100	9.56	93.1	4.91
ethion	1.65	8.00–8000	0.9985	73.9	29.8	80.2	17.7	83.4	18.6
endosulfan II	16.1	40.0–8000	0.9939	NA	NA	86.8	15.3	91.1	7.58
4,4′-DDD	1.23	8.00–8000	0.9961	96.5	17.8	100	19.3	104	10.9
2,2’,4,4′,5,5′-hexachlorobiphenyl	0.668	8.00–8000	0.9953	95.6	3.32	98.5	9.83	94.2	7.64
norflurazon	3.92	8.00–8000	0.9946	83.4	15.1	93.5	13.4	90.1	6.53
butyl benzyl phthalate	3.39	8.00–8000	0.9978	113	28.3	110	13.2	103	6.11
endosulfan sulfate	9.06	20.0–8000	0.9939	NA	NA	85.6	11.9	81.3	7.61
4,4′-DDT	3.57	8.00–8000	0.9975	87.8	25.4	98.6	23.5	81.8	10.4
hexazinone	16.3	40.0–8000	0.9978	NA	NA	80.1	10.5	85.7	6.97
2,2’,3,4,4′,5′-hexachlorobiphenyl	0.706	8.00–8000	0.9951	94.2	3.15	92.9	8.16	92.4	5.74
di(2-ethylhexyl)adipate	12.5	20.0–8000	0.9966	NA	NA	102	15.4	101	18.2
tebuconazole	9.44	20.0–8000	0.9939	NA	NA	100	8.68	95.9	3.92
methoxychlor	1.90	8.00–8000	0.9919	77.8	19.6	81.5	11.1	77.3	16.1
benzo[*a*]anthracene	3.42	8.00–8000	0.9978	89.4	12.0	94.9	17.4	93.8	10.3
chrysene	3.23	8.00–8000	0.9983	95.7	15.9	96.1	13.0	93.9	8.48
2,2’,3,4,4′,5,5′-heptachlorobiphenyl	1.03	8.00–8000	0.9939	89.3	7.81	96.5	15.1	97.6	14.8
di(2-ethylhexyl)phthalate	17.6	40.0–8000	0.9962	NA	NA	116	20.7	119	22.9
fenarimol	17.4	8.00–8000	0.9984	NA	NA	88.5	7.11	90.9	9.79
cis-permethrin	2.45	8.08–8000	0.9906	104	27.2	96.3	11.1	94.4	8.88
trans-permethrin	1.96	8.00–8000	0.9967	87.7	25.3	98.7	14.2	94.9	13.9
benzo[*b*]fluorancene	2.73	8.00–8000	0.9986	87.0	18.5	96.0	14.1	89.3	10.4
benzo[k]fluorancene	3.21	8.00–8000	0.9963	85.0	17.0	94.9	20.0	91.2	15.0
fluridone	3.84	8.00–8000	0.9983	70.9	26.4	77.0	14.3	76.8	15.9
benzo[*a*]pyrene	16.2	8.00–8000	0.9984	NA	NA	90.9	15.3	86.9	7.83
dibenzo[a,h]anthracene	3.21	8.00–8000	0.9960	83.0	24.0	91.0	6.27	84.7	10.5
indeno[1,2,3-*c*,*d*]pyrene	1.33	8.00–8000	0.8885	87.5	11.0	84.1	10.1	85.6	11.6
benzo[g,h,i]perylene	2.78	8.00–8000	0.9910	95.1	10.3	101	13.3	98.1	10.9

A recovery test was used to evaluate accuracy and precision of the method. Three groups of deionized water samples free of analytes of interest were spiked with the working standard to achieve concentrations of 12, 60, and 300 ng/L, respectively. These fortified water samples were then analyzed with the optimized method. Recovery of the surrogates was in a range of 80–120% to verify that the extraction and desorption occurred properly for each sample. Recoveries and RSDs of each analyte of interest were calculated at each fortified concentration. For those compounds detectable at 12 ng/L, the mean recovery was 95.7%, with an RSD of 15.3%. As 12 ng/L is close to the LODs of some analytes, relatively higher RSDs were expected. For example, *4*,*4’-*dichlorodiphenyltrichloroethane (4,4′-DDT), acetochlor, butylated hydroxytoluene (BHT), butyl benzyl phthalate, cis-permethrin, ethion, Fluridone, and trifluralin had greater than 25% RSDs at this low concentration level. However, none of them had RSDs over 30%. At 60 and 300 ng/L, the mean recovery for the analytes was 95.9% and 93.1%, respectively, with RSDs of 11.3 and 9.50%, respectively. 117 of the 123 target compounds were quantifiable at 60 ng/L. Recoveries of most analytes were in a range of 80–120%. Phosphamidon was the only analyte that had a recovery lower than 70% and an RSD higher than 30%. As discussed earlier, further approaches will be investigated to improve the results of these compounds. The detailed results are shown in Table 1. A comparison of figures of merit of previous studies using SPME and SBSE coupled with GC–QMS is provided in Table S2 in the Supporting Information.

A room-temperature storage stability test was performed with a holding time of 1, 4, 7, and 10 days after the samples were extracted with FEVE and stored in the FSPs with sleeves. The recoveries of OCPs, ONPs, OPPs, OSPs, phthalates and others, PAHs, and PCBs at day 1, day 4, day 7, and day 10 were compared with those at day 0, where the FSPs were analyzed immediately after the extraction. These relative recoveries are shown in Figure S4 in the Supporting Information. At day 1, all categories had recoveries over 97%, relative to those at day 0. The recoveries started declining as the storage time increased. Nevertheless, all categories were still able to hold at least 90% recovery at day 7. At day 10, OCPs, PAHs, and PCBs dropped to between 85 and 89%, and the other categories were in a range of 91–95%. Overall, all the analytes showed adequate storage stabilities when stored in sleeve-isolated FSPs after FEVE. This result opens up the potential for this technique to be utilized in extraction stations or laboratories with no access to GC–MS instruments, where FEVE can be performed off-line, and after extraction, the FSPs with sleeves can be shipped nationally or even internationally to analytical laboratories for TD–GC–MS analysis.

### Analysis of Drinking Water and Surface Water Samples

After method development, the FEVE–TD–GC–MS method was employed to analyze 10 drinking water and surface water samples. Samples A, B, C, and D were four different brands of commercially available bottled water obtained from local supermarkets in Simi Valley, CA. Sample E was tap water; F, G, and I were creek water samples; and H and J were lake water samples. All these sampling sites are located in Ventura County and Los Angeles County, CA. Overall, more target analytes were found in the surface water samples than in the drinking water samples. Creek water F had 38 analytes of interest detected, which was the most among all the samples. 23 out of 123 target compounds were detected in bottled water D, which was the highest number among all the drinking water samples. Heptachlor epoxide, chlorpyrifos, metolachlor, butachlor, and 2,3′,4′,5-tetrachlorobiphenyl were the most frequently detected compounds. They were found in 4 out of the 10 water samples. The highest concentration of all compounds detected was that of bromacil at 1986 ng/L in creek water G. It was noticed that significantly more analytes were found in creek water samples than in drinking water or lake water samples. The average number of analytes detected in creek water, drinking water, and lake water samples was 30, 10, and 7, respectively. Bottled water A was found to contain 2,6-dinitrotoluene, at a concentration of 135 ng/L. Heptachlor epoxide was detected in bottled water B and D, at concentrations of 60.7 and 87.5 ng/L, respectively. Bottled water D also contained endrin, atrazine, propazine, g-HCH, and chrysene, in a concentration range of 72.0–180 ng/L. Bottled water C had dibutyl phthalate, chlorpyrifos, and nitrofen detected, at concentrations of 899, 82.4, and 314 ng/L, respectively. These chemicals are listed in California Proposition 65 for potential cancer, developmental, and reproductive toxicity. However, none of these compounds detected exceeded the maximum allowable dose level set by the existing regulations. The detailed results are provided in Table S4 in the Supporting Information.

## Conclusions

In this work, FEVE, a quantitative and green approach, was designed, developed, evaluated, and applied to analysis of 123 SVOCs in drinking water and surface water samples. This method enables quantification of a broad range of semivolatile compounds simultaneously, meanwhile providing a high level of sensitivity, accuracy, and precision. The extraction and analysis process is highly automated, enabling a simple and efficient workflow for analytical laboratories, and completely eliminates the use of solvents during sampling, analysis, and cleanup. Besides drinking water and surface water, FEVE also has the potential to be applied to other matrices. For example, for analysis of more complex environmental, biological, and foodstuff samples, after a simple pre-extraction to remove suspendid solids in the samples, a mixture of water and extract can be analyzed using FEVE. A 6 mL version of the FEVE technology will also be available to analyze up to 5 mL of the sample, which can further lower the detection limits.

## Supporting Information Available

Additional experimental details, analyte information, and method development and sample analysis results (PDF)

## References

[ref1] Arthur C. L.; Pawliszyn J. Solid phase microextraction with thermal desorption using fused silica optical fibers. Anal. Chem. 1990, 62, 2145–2148. 10.1021/ac00218a019.

[ref2] Baltussen E.; Sandra P.; David F.; Cramers C. Stir bar sorptive extraction (SBSE), a novel extraction technique for aqueous samples: theory and principles. J. Microcolumn Sep. 1999, 11, 737–747. 10.1002/(sici)1520-667x(1999)11:10<737::aid-mcs7>3.0.co;2-4.

[ref3] Dietz C.; Sanz J.; Cámara C. Recent developments in solid-phase microextraction coatings and related techniques. J. Chromatogr. A 2006, 1103, 183–192. 10.1016/j.chroma.2005.11.041. 16337213

[ref4] Spietelun A.; Pilarczyk M.; Kloskowski A.; Namieśnik J. Current trends in solid-phase microextraction (SPME) fibre coatings. Chem. Soc. Rev. 2010, 39, 4524–4537. 10.1039/c003335a. 20882243

[ref5] Merkle S.; Kleeberg K. K.; Fritsche J. Recent developments and applications of solid phase microextraction (SPME) in food and environmental analysis—a review. Chromatography 2015, 2, 293–381. 10.3390/chromatography2030293.

[ref6] Piri-Moghadam H.; Alam M. N.; Pawliszyn J. Review of geometries and coating materials in solid phase microextraction: opportunities, limitations, and future perspectives. Anal. Chim. Acta 2017, 984, 42–65. 10.1016/j.aca.2017.05.035. 28843569

[ref7] Jalili V.; Barkhordari A.; Ghiasvand A. A comprehensive look at solid-phase microextraction technique: A review of reviews. Microchem. J. 2020, 152, 104319 10.1016/j.microc.2019.104319.

[ref8] David F.; Sandra P. Stir bar sorptive extraction for trace analysis. J. Chromatogr. A 2007, 1152, 54–69. 10.1016/j.chroma.2007.01.032. 17239895

[ref9] Prieto A.; Basauri O.; Rodil R.; Usobiaga A.; Fernández L.; Etxebarria N.; Zuloaga O. Stir-bar sorptive extraction: A view on method optimisation, novel applications, limitations and potential solutions. J. Chromatogr. A 2010, 1217, 2642–2666. 10.1016/j.chroma.2009.12.051. 20083248

[ref10] David F.; Ochiai N.; Sandra P. Two decades of stir bar sorptive extraction: A retrospective and future outlook. TrAC, Trends Anal. Chem. 2019, 112, 102–111. 10.1016/j.trac.2018.12.006.

[ref11] Anastassiades M.; Scherbaum E. Sample handling and clean-up procedures II—new developments. Compr. Anal. Chem. 2005, 43, 113–233. 10.1016/s0166-526x(05)80024-8.

[ref12] Hao W.; Dillard A.; Macherone A.; Stuff J.; Pamuku M. Quantification of persistent organic pollutants in human whole blood samples using stir bar sorptive extraction coupled with GC/MS/MS and isotope dilution mass spectrometry. Microchem. J. 2019, 153, 104279 10.1080/19440049.2020.1749315.

[ref13] Hao W.; Kingston H. S.; Dillard A.; Stuff J.; Pamuku M. Quantification of persistent organic pollutants in dietary supplements using stir bar sorptive extraction coupled with GC-MS/MS and isotope dilution mass spectrometry. Food Addit. Contam., Part A: Chem., Anal., Control, Exposure Risk Assess. 2020, 37, 1202–1215. 10.1080/19440049.2020.1749315. 32364029

[ref14] Gilart N.; Marcé R. M.; Borrull F.; Fontanals N. New coatings for stir-bar sorptive extraction of polar emerging organic contaminants. TrAC, Trends Anal. Chem. 2014, 54, 11–23. 10.1016/j.trac.2013.10.010.

[ref15] Camino-Sánchez F. J.; Rodríguez-Gómez R.; Zafra-Gómez A.; Santos-Fandila A.; Vílchez J. L. Stir bar sorptive extraction: recent applications, limitations and future trends. Talanta 2014, 130, 388–399. 10.1016/j.talanta.2014.07.022. 25159426

[ref16] Pfannkoch E. A.; Stuff J. R.; Whitecavage J. A.; Blevins J. M.; Seely K. A.; Moran J. H. A high throughput method for measuring polycyclic aromatic hydrocarbons in seafood using QuEChERS extraction and SBSE. Int. J. Anal. Chem. 2015, 2015, 359629 10.1155/2015/359629. 25873967 PMC4383357

[ref17] Trujillo-Rodríguez M. J.; Anderson J. L.; Dunham S. J.; Noad V. L.; Cardin D. B. Vacuum-assisted sorbent extraction: An analytical methodology for the determination of ultraviolet filters in environmental samples. Talanta 2020, 208, 120390 10.1016/j.talanta.2019.120390. 31816753

[ref18] Jeleń H. H.; Gaca A.; Marcinkowska M. Use of sorbent-based vacuum extraction for determination of volatile phenols in beer. Food Anal. Methods 2018, 11, 3089–3094. 10.1007/s12161-018-1277-z.

[ref19] Crucello J.; Medeiros Junior I.; Mesquita de Carvalho R.; Wang Hantao L. Profiling organic acids in produced water samples using vacuum-assisted sorbent extraction and gas chromatography coupled to Fourier transform Orbitrap mass spectrometry. Microchem. J. 2022, 180, 107581 10.1016/j.microc.2022.107581.

[ref20] EPA Method 525.3 - Determination of Semivolatile Organic Chemicals in Drinking Water by Solid Phase Extraction and Capillary Column Gas Chromatography/Mass Spectrometry (GC/MS). 2014 (accessed 5/1/2022).

[ref21] Ravindra K.; Sokhi R.; Vangrieken R. Atmospheric polycyclic aromatic hydrocarbons: source attribution, emission factors and regulation. Atmos. Environ. 2008, 42, 2895–2921. 10.1016/j.atmosenv.2007.12.010.

[ref22] Chen B.; Xuan X.; Zhu L.; Wang J.; Gao Y.; Yang K.; Shen X.; Lou B. Distributions of polycyclic aromatic hydrocarbons in surface waters, sediments and soils of Hangzhou City, China. Water Res. 2004, 38, 3558–3568. 10.1016/j.watres.2004.05.013. 15325182

[ref23] Schecter A.; Colacino J.; Haffner D.; Patel K.; Opel M.; Päpke O.; Birnbaum L. Perfluorinated compounds, polychlorinated biphenyls, and organochlorine pesticide contamination in composite food samples from Dallas, Texas, USA. Environ. Health Perspect. 2010, 118, 796–802. 10.1289/ehp.0901347. 20146964 PMC2898856

[ref24] Colosio C.; Tiramani M.; Maroni M. Neurobehavioral effects of pesticides: state of the art. Neurotoxicology 2003, 24, 577–591. 10.1016/S0161-813X(03)00055-X. 12900071

[ref25] Safe S. H. Polychlorinated biphenyls (PCBs): environmental impact, biochemical and toxic responses, and implications for risk assessment. Crit. Rev. Toxicol. 1994, 24, 87–149. 10.3109/10408449409049308. 8037844

[ref26] Tankiewicz M.; Fenik J.; Biziuk M. Determination of organophosphorus and organonitrogen pesticides in water samples. TrAC, Trends Anal. Chem. 2010, 29, 1050–1063. 10.1016/j.trac.2010.05.008.

[ref27] Wong J. W.; Webster M. G.; Halverson C. A.; Hengel M. J.; Ngim K. K.; Ebeler S. E. Multiresidue pesticide analysis in wines by solid-phase extraction and capillary gas chromatography– mass spectrometric detection with selective ion monitoring. J. Agric. Food Chem. 2003, 51, 1148–1161. 10.1021/jf0209995. 12590449

[ref28] Bansal V.; Kim K.-H. Review of PAH contamination in food products and their health hazards. Environ. Int. 2015, 84, 26–38. 10.1016/j.envint.2015.06.016. 26203892

[ref29] Kwong T. C. Organophosphate pesticides: biochemistry and clinical toxicology. Ther. Drug Monit. 2002, 24, 144–149. 10.1097/00007691-200202000-00022. 11805735

[ref30] Kamrin M. A. Phthalate risks, phthalate regulation, and public health: a review. J. Toxicol. Environ. Health, Part B Rev. 2009, 12, 157–174. 10.1080/10937400902729226. 19235623

[ref31] Hook G. L.; Kimm G.; Betsinger G.; Savage P. B.; Swift A.; Logan T.; Smith P. A. Solid phase microextraction sampling and gas chromatography/mass spectrometry for field detection of the chemical warfare agent O-ethyl S-(2-diisopropylaminoethyl) methylphosphonothiolate (VX). J. Sep. Sci. 2003, 26, 1091–1096. 10.1002/jssc.200301561.

[ref32] Kim Y.-H.; Kim K.-H.; Szulejko J. E.; Parker D. Development of the detection threshold concept from a close look at sorption occurrence inside a glass vial based on the in-vial vaporization of semivolatile fatty acids. Anal. Chem. 2014, 86, 6640–6647. 10.1021/ac501382e. 24881858

[ref33] Abdul-Fattah A. M.; Oeschger R.; Roehl H.; Bauer Dauphin I. B.; Worgull M.; Kallmeyer G.; Mahler H.-C. Investigating factors leading to fogging of glass vials in lyophilized drug products. Eur. J. Pharm. Biopharm. 2013, 85, 314–326. 10.1016/j.ejpb.2013.06.007. 23791681

[ref34] Huang Y.; Hu Y.; Yuill E. M.; Marriott A. S.; Chadwick J.; Li J.; Zang J.; Miller S. A. Circumventing glass vial and diluent effects on solution stability of small molecule analytes during analytical method development and validation. J. Pharm. Biomed. Anal. 2022, 213, 114676 10.1016/j.jpba.2022.114676. 35240407

[ref35] Lambropoulou D. A.; Albanis T. A. Optimization of headspace solid-phase microextraction conditions for the determination of organophosphorus insecticides in natural waters. J. Chromatogr. A 2001, 922, 243–255. 10.1016/s0021-9673(01)00953-0. 11486869

[ref36] Helaleh M. I.; Fujii S.; Korenaga T. Column silylation method for determining endocrine disruptors from environmental water samples by solid phase micro-extraction. Talanta 2001, 54, 1039–1047. 10.1016/s0039-9140(01)00386-1. 18968325

[ref37] Carabias-Martínez R.; García-Hermida C.; Rodríguez-Gonzalo E.; Ruano-Miguel L. Behaviour of carbamate pesticides in gas chromatography and their determination with solid-phase extraction and solid-phase microextraction as preconcentration steps. J. Sep. Sci. 2005, 28, 2130–2138. 10.1002/jssc.200400047. 16318209

[ref38] Djozan D.; Mahkam M.; Ebrahimi B. Preparation and binding study of solid-phase microextraction fiber on the basis of ametryn-imprinted polymer: application to the selective extraction of persistent triazine herbicides in tap water, rice, maize and onion. J. Chromatogr. A 2009, 1216, 2211–2219. 10.1016/j.chroma.2008.12.101. 19185305

[ref39] Djozan D.; Ebrahimi B. Preparation of new solid phase micro extraction fiber on the basis of atrazine-molecular imprinted polymer: application for GC and GC/MS screening of triazine herbicides in water, rice and onion. Anal. Chim. Acta 2008, 616, 152–159. 10.1016/j.aca.2008.04.037. 18482598

[ref40] Filho A.; dos Santos F. N.; Pereira P. A. d. P. Development, validation and application of a method based on DI-SPME and GC–MS for determination of pesticides of different chemical groups in surface and groundwater samples. Microchem. J. 2010, 96, 139–145. 10.1016/j.microc.2010.02.018.

[ref41] Magdic S.; Pawliszyn J. B. Analysis of organochlorine pesticides using solid-phase microextraction. J. Chromatogr. A 1996, 723, 111–122. 10.1016/0021-9673(95)00857-8. 8819826

[ref42] Menezes Filho A.; dos Santos F. N.; Pereira P. A. Development, validation and application of a methodology based on solid-phase micro extraction followed by gas chromatography coupled to mass spectrometry (SPME/GC–MS) for the determination of pesticide residues in mangoes. Talanta 2010, 81, 346–354. 10.1016/j.talanta.2009.12.008. 20188930

[ref43] Nakamura S.; Daishima S. Simultaneous determination of 64 pesticides in river water by stir bar sorptive extraction and thermal desorption-gas chromatography-mass spectrometry. Anal. Bioanal. Chem. 2005, 382, 99–107. 10.1007/s00216-005-3158-8. 15900458

[ref44] Ochiai N.; Sasamoto K.; Kanda H.; Yamagami T.; David F.; Tienpont B.; Sandra P. Optimization of a multi-residue screening method for the determination of 85 pesticides in selected food matrices by stir bar sorptive extraction and thermal desorption GC–MS. J. Sep. Sci. 2005, 28, 1083–1092. 10.1002/jssc.200500017. 16013835

[ref45] Pérez-Carrera E.; León V. M.; Parra A. G.; González-Mazo E. Simultaneous determination of pesticides, polycyclic aromatic hydrocarbons and polychlorinated biphenyls in seawater and interstitial marine water samples, using stir bar sorptive extraction–thermal desorption–gas chromatography–mass spectrometry. J. Chromatogr. A 2007, 1170, 82–90. 10.1016/j.chroma.2007.09.013. 17915232

[ref46] Ochiai N.; Sasamoto K.; Ieda T.; David F.; Sandra P. Multi-stir bar sorptive extraction for analysis of odor compounds in aqueous samples. J. Chromatogr. A 2013, 1315, 70–79. 10.1016/j.chroma.2013.09.070. 24094753

[ref47] Sandra P.; Tienpont B.; David F. Multi-residue screening of pesticides in vegetables, fruits and baby food by stir bar sorptive extraction–thermal desorption–capillary gas chromatography–mass spectrometry. J. Chromatogr. A 2003, 1000, 299–309. 10.1016/s0021-9673(03)00508-9. 12877176

[ref48] Farajzadeh M. A.; Djozan D.; Nouri N.; Bamorowat M.; Shalamzari M. S. Coupling stir bar sorptive extraction-dispersive liquid–liquid microextraction for preconcentration of triazole pesticides from aqueous samples followed by GC-FID and GC-MS determinations. J. Sep. Sci. 2010, 33, 1816–1828. 10.1002/jssc.201000088. 20449842

[ref49] Rodil R.; Popp P. Development of pressurized subcritical water extraction combined with stir bar sorptive extraction for the analysis of organochlorine pesticides and chlorobenzenes in soils. J. Chromatogr. A 2006, 1124, 82–90. 10.1016/j.chroma.2006.05.028. 16765970

[ref50] Grossi P.; Olivares I. R.; de Freitas D. R.; Lancas F. M. A novel HS-SBSE system coupled with gas chromatography and mass spectrometry for the analysis of organochlorine pesticides in water samples. J. Sep. Sci. 2008, 31, 3630–3637. 10.1002/jssc.200800338. 18850636

