# Supplementary material for: Full Evaporative Vacuum Extraction—A Quantitative and Green Approach for Analysis of Semivolatile Organic Compounds in Drinking Water and Surface Water Using GC–MS
Source: Anal Chem. 2023 Feb 7;95(8):3959–67. doi: 10.1021/acs.analchem.2c03414 (PMC9979150; doi:10.1021/acs.analchem.2c03414)
Supplement: Supplementary file 1 — ac2c03414_si_001.pdf [file ac2c03414_si_001.pdf]

Supporting Information

Full Evaporative Vacuum Extraction – A Quantitative and Green Approach for Analysis of Semivolatile Organic Compounds in Drinking Water and Surface Water Using GC-MS

Weier Hao*, Daniel B. Cardin

Entech Instruments Inc, 2207 Agate Ct, Simi Valley, CA, 93065, USA

Corresponding Author Email: weier.hao@gmail.com

Contents:

Figure S1. Rendering of the FEVE instrument mounted on a vacuum extraction bar with 30 samples loaded.

Figure S2. Valve controls and flow directions of four GC-MS analysis stages: Idle/Preheat (a), Desorption (b), Splittless Transfer/Bakeout (c), and Backflush (d). Column 1: Quadrex UAC-1MS (5 m × 0. 53 mm × 0.15 µm). Column 2: Agilent HP-5MS (30 m × 0.25 mm × 0.5 µm).

Figure S3. Recovery of OCPs, ONPs, OPPs, OSPs, phthalates and others, PAHs, and PCBs with different desorption temperatures (n=3).

Figure S4. Recovery of OCPs, ONPs, OPPs, OSPs, phthalates and others, PAHs, and PCBs in FSPs with sleeves 1, 4, 7, and 10 days after FEVE, relative to the recovery of these analytes analyzed immediately after FEVE (n=3).

Figure S5. Total Ion Chromatogram of the 123 target analytes at a concentration of 4000 ng/L.

Figure S6. Examples of Selected Ion Monitoring chromatograms of quantification and confirmation ions of 2,2',5-Trichlorobiphenyl (a), Heptachlor (b), and Dacthal (c) at a concentration of 8 ng/L.

Figure S7. Sampling locations for water samples D-J in Ventura County and Los Angeles County, CA.

Table S1. Chemical name, CAS number, category, GC-MS retention time, quantitation and confirmation ions, and method detection limit (MDL) of the target SVOC analytes.

Table S2. Comparisons of LODs, errors, and RSDs of previous studies using SPME and SBSE coupled with GC-MS.

Table S3. Recovery of individual analytes in FSPs with sleeves 1, 4, 7, and 10 days after FEVE, relative to the recovery of these analytes analyzed immediately after FEVE (n=3).

Table S4. Measurements of 123 target SVOCs in 10 drinking water and surface water samples (n=3). A, B, C, and D were bottled water samples; E was tap water sample; F, G, and I were Creek water samples; and H and J were lake water samples.

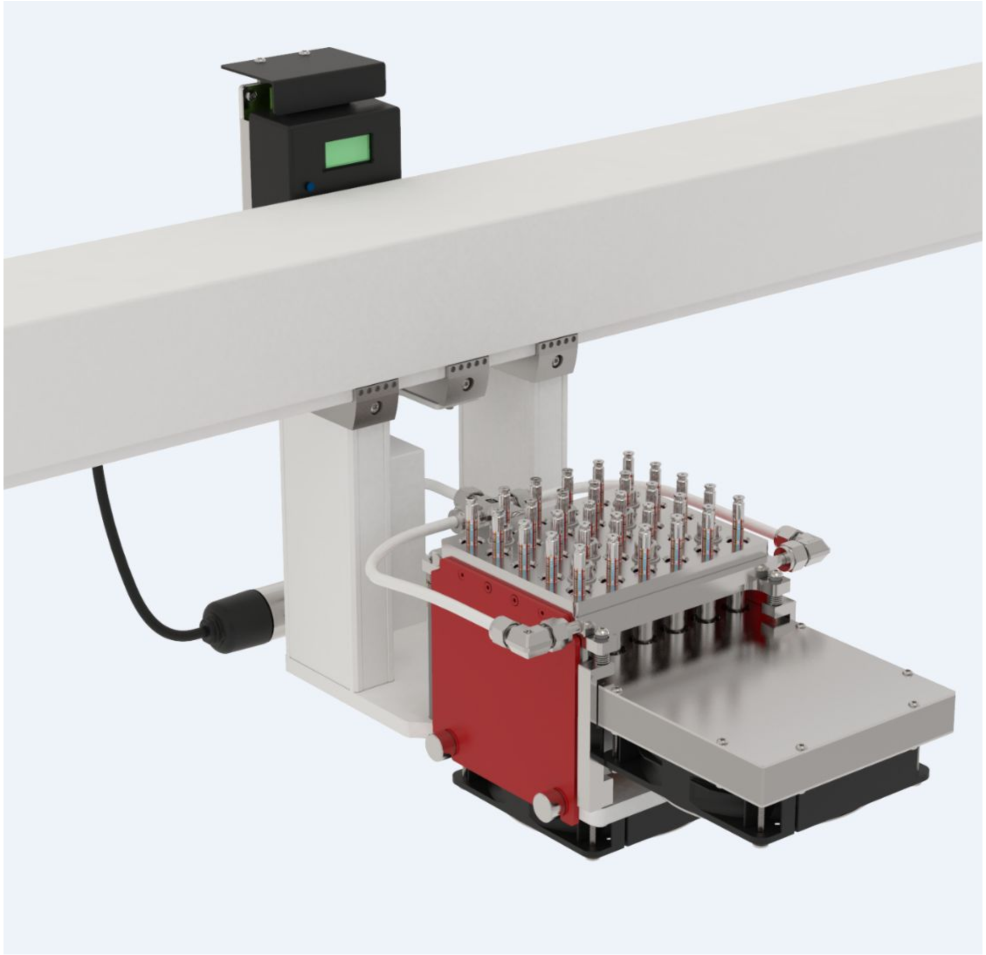

Figure S1. Rendering of the FEVE instrument mounted on a vacuum extraction bar with 30 samples loaded.

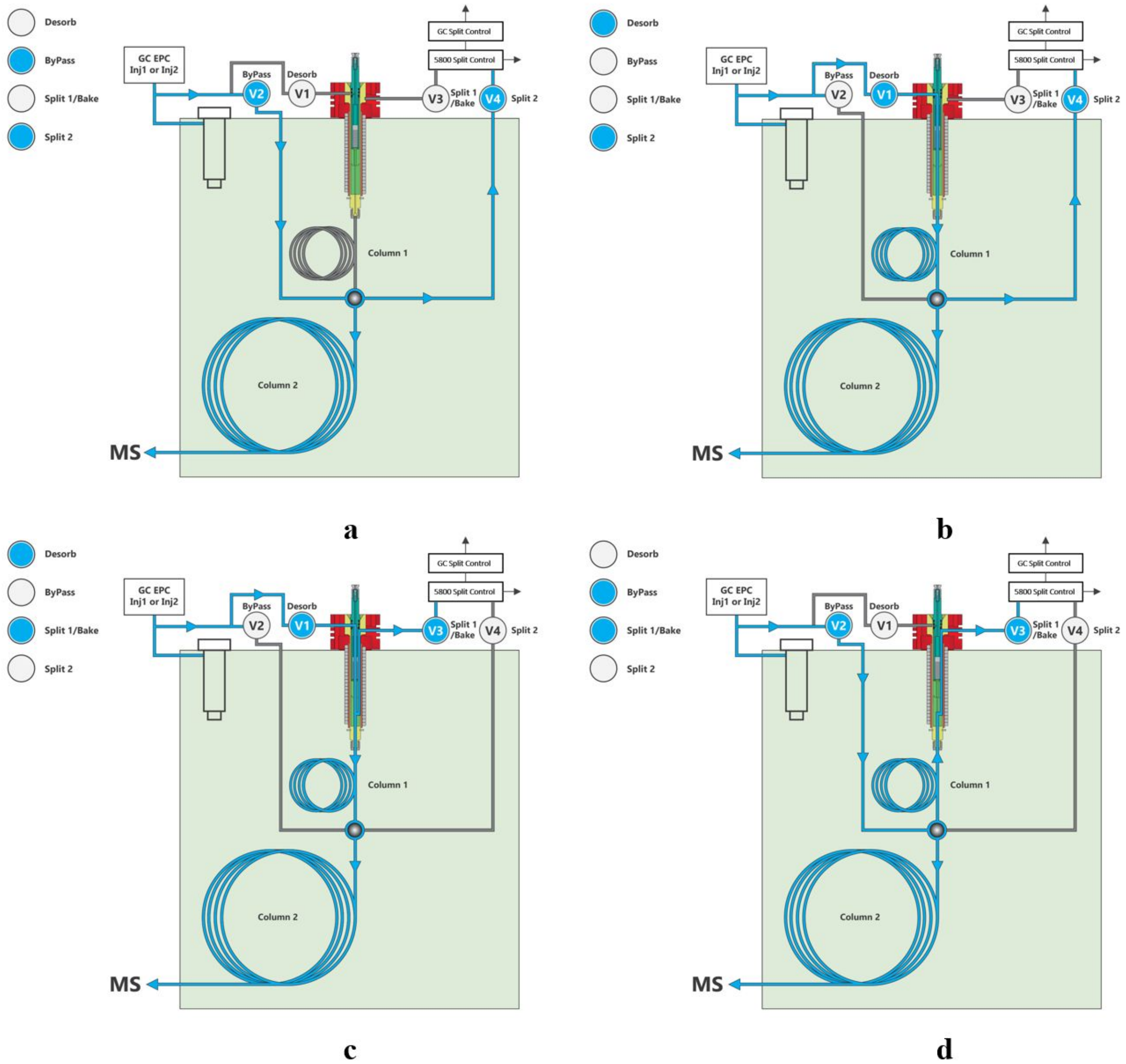

Figure S2. Valve controls and flow directions of four GC-MS analysis stages: Idle/Preheat (a), Desorption (b), Splittless Transfer/Bakeout (c), and Backflush (d). Column 1: Quadrex UAC-1MS (5 m × 0. 53 mm × 0.15 µm). Column 2: Agilent HP-5MS (30 m × 0.25 mm × 0.5 µm).

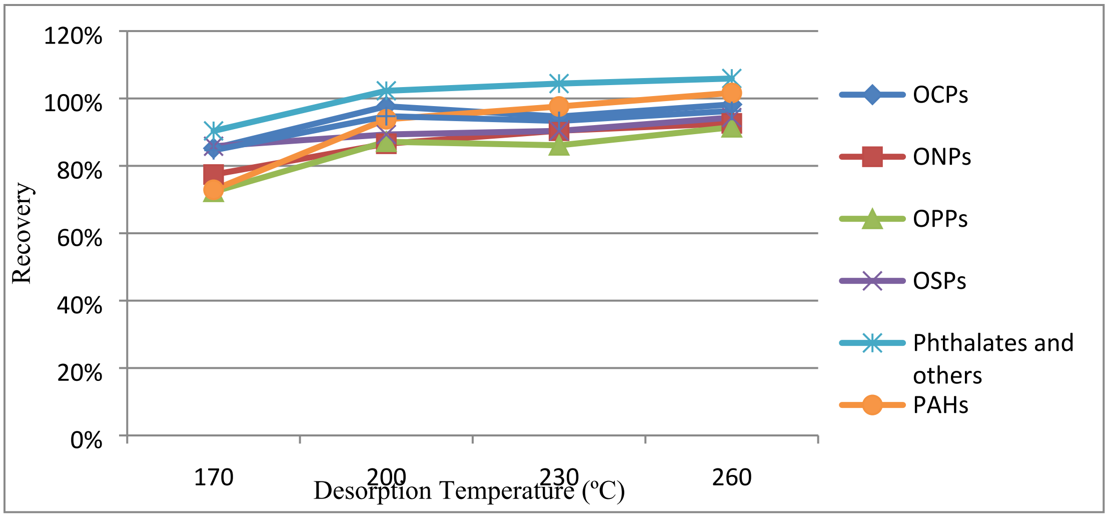

Figure S3. Recovery of OCPs, ONPs, OPPs, OSPs, phthalates and others, PAHs, and PCBs with different desorption temperatures (n=3).

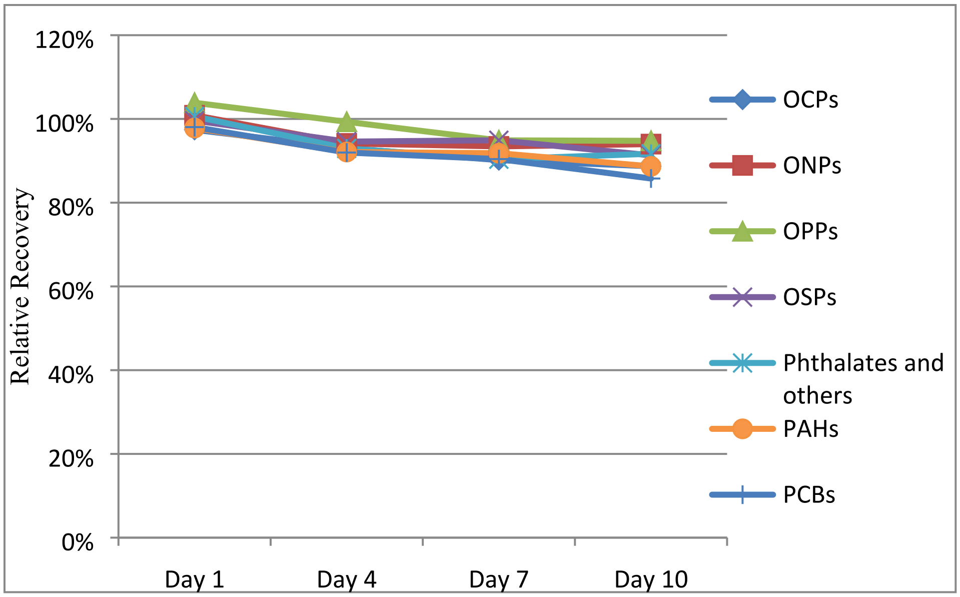

Figure S4. Recovery of OCPs, ONPs, OPPs, OSPs, phthalates and others, PAHs, and PCBs in FSPs with sleeves 1, 4, 7, and 10 days after FEVE, relative to the recovery of these analytes analyzed immediately after FEVE (n=3).

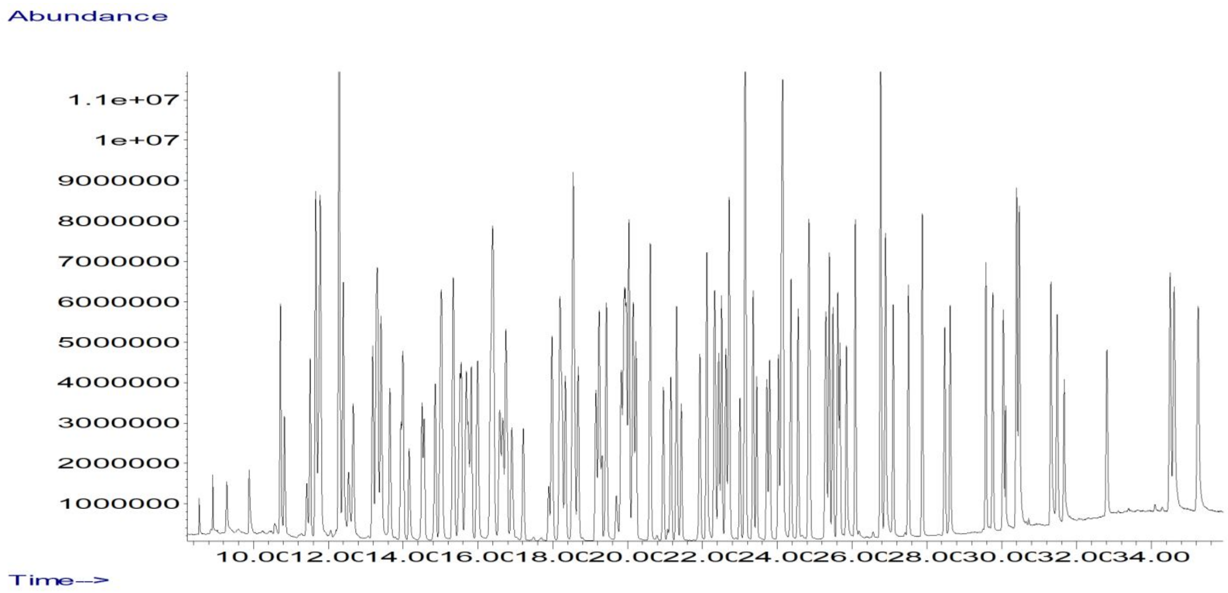

Figure S5. Total Ion Chromatogram of the 123 target analytes at a concentration of 4000 ng/L.

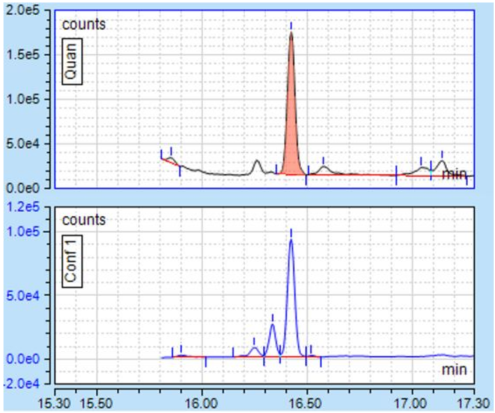

(a)

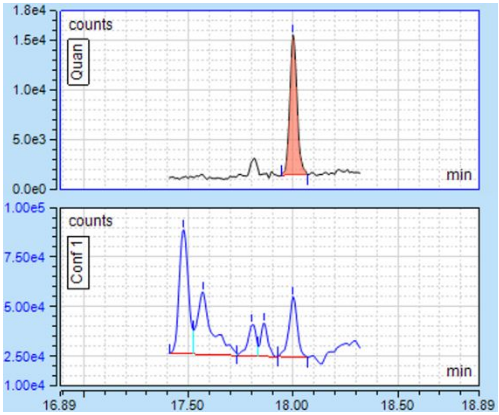

(b)

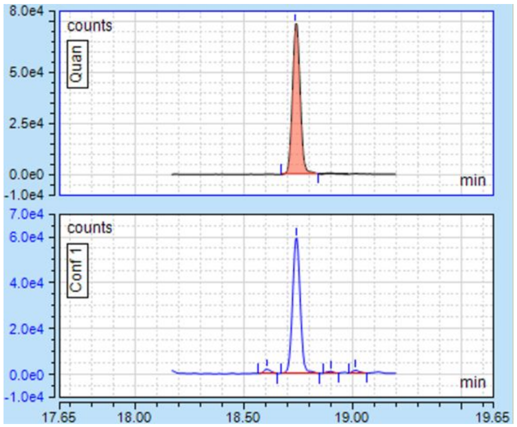

(c)

Figure S6. Examples of Selected Ion Monitoring chromatograms of quantification and confirmation ions of 2,2',5-Trichlorobiphenyl (a), Heptachlor (b), and Dacthal (c) at a concentration of 8 ng/L.

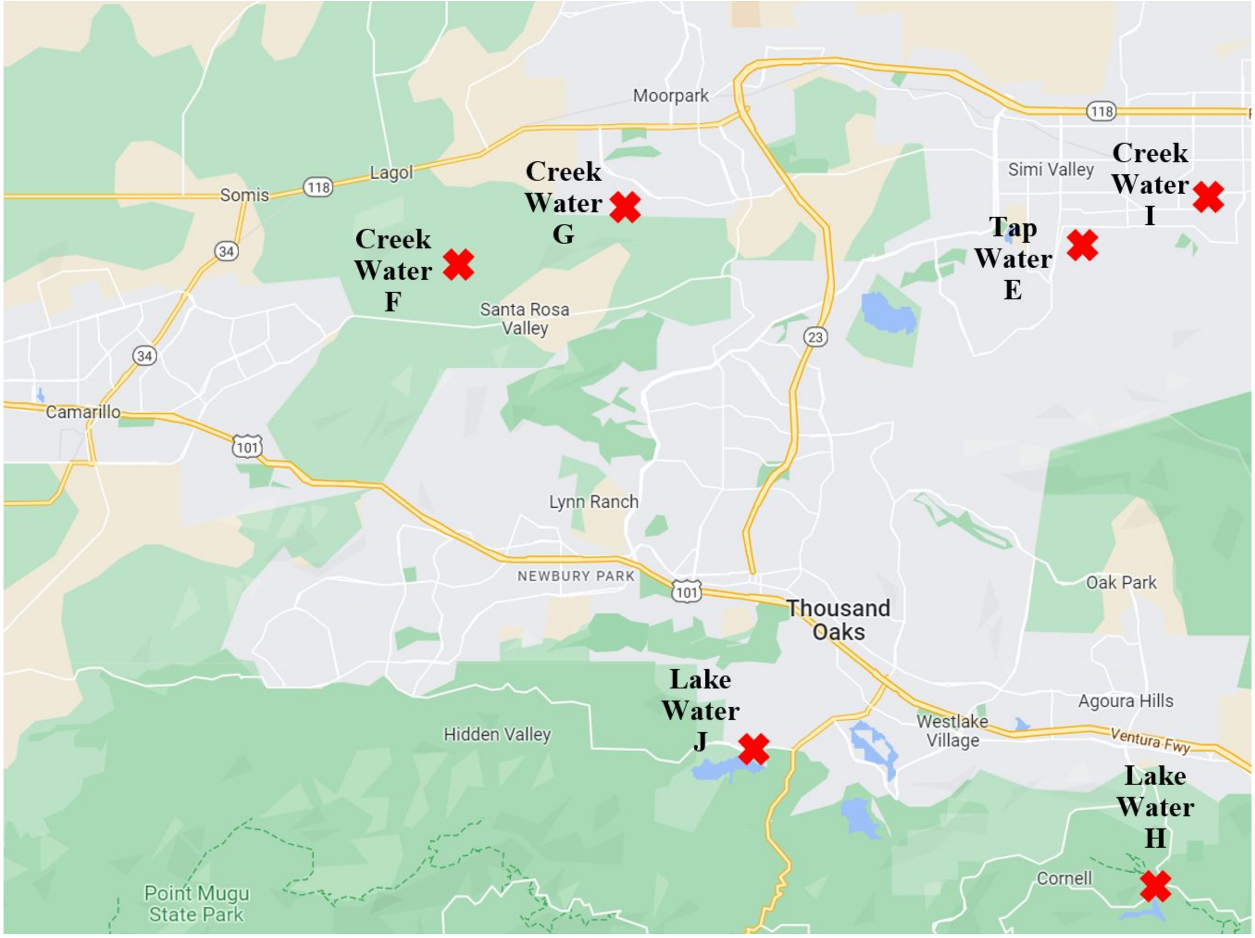

Figure S7. Sampling locations for water samples E-J in Ventura County and Los Angeles County, CA.

Table S1. Chemical name, CAS number, category, GC-MS retention time, quantitation and confirmation ions, and method detection limit (MDL) of the target SVOC analytes.

Chemical Name	CAS Number	Category*	RT (min)	QI (m/z)	CI (m/z)	MDL (ng/L)
DIMP	1445-75-6	c	8.60	97	123	82.5
Isophorone	78-59-1	e	9.26	82	138	18.8
1,3-Dimethyl-2-nitrobenzene	81-20-9	h	9.92	134	79	NA
Dichlorvos	62-73-7	c	10.41	109	85	60.0
HCCPD	77-47-4	a	11.43	237	235	22.7
EPTC	759-94-4	d	11.51	128	86	47.2
Mevinphos	7786-34-7	c	12.07	127	192	24.0
Butylate	2008-41-5	d	12.22	146	57	13.2
Dimethylphthalate	131-11-3	e	12.38	163	77	22.1
Vernolate	1929-77-7	c	12.38	128	86	18.1
2,6-Dinitrotoluene	121-14-2	b	12.43	165	89	22.6
Etridiazole	2593-15-9	d	12.48	211	183	12.2
Pebulate	1114-71-2	d	12.52	128	72	31.0
Acenaphthylene	208-96-8	f	12.69	152	151	14.1
Chlorneb	2675-77-6	a	12.97	191	193	52.4
BHT	128-37-0	e	13.04	205	220	15.3
2-Chlorobiphenyl	2051-60-7	g	13.09	188	152	3.53
Tebuthiuron	34014-18-1	d	13.19	156	171	27.3
2,4-Dinitrotoluene	121-14-2	b	13.25	165	89	74.6
Molinate	2212-67-1	d	13.47	126	55	8.19
DEET	134-62-3	b	13.78	119	190	39.2
Diethylphthalate	84-66-2	e	13.84	149	177	93.7
4-Chlorobiphenyl	2051-62-9	g	14.00	188	152	3.76
Fluorene	86-73-7	f	14.15	166	165	21.0
Propachlor	1918-16-7	b	14.16	120	170	70.6
Ethoprop	13194-48-4	c	14.42	158	97	24.4
Cycloate	1134-23-2	d	14.51	83	154	31.9
Chlorpropham	101-21-3	b	14.61	127	213	24.6
Trifluralin	1582-09-8	b	14.65	306	264	4.71
Phorate	298-02-2	c	15.13	75	121	26.5
a-HCH	319-84-6	a	15.34	181	183	16.2
2,4'-Dichlorobiphenyl	34883-43-7	g	15.38	222	152	4.48
Atraton	1610-17-9	b	15.45	196	211	5.30
Hexachlorobenzene	118-74-1	a	15.55	284	142	18.4

Prometon	1610-18-0	b	15.57	210	225	14.1
Simazine	122-34-9	b	15.59	201	186	20.3
Dimethipin	55290-64-7	d	15.65	54	53	41.2
Atrazine	1912-24-9	b	15.69	200	215	4.37
Propazine	139-40-2	b	15.77	214	229	4.81
b-HCH	319-85-7	a	15.8	181	183	23.1
Pentachlorophenol	87-86-5	a	16.01	266	268	45.4
d-HCH	319-86-8	a	16.09	181	183	20.2
Pronamide	23950-58-5	b	16.16	173	175	18.3
2,2',5-Trichlorobiphenyl	37680-65-2	g	16.34	186	256	7.54
Terbacil	5902-51-2	b	16.44	161	117	49.7
Chlorothalonil	1897-45-6	b	16.46	266	268	17.7
Disulfoton	298-04-4	d	16.49	88	97	19.1
Phenanthrene	85-01-8	f	16.55	178	176	11.4
g-HCH	58-89-9	a	16.58	181	183	22.5
Anthracene	120-12-7	f	16.68	178	176	25.3
Phosphamidon	13171-21-6	c	17.10	127	264	197
Acetochlor	34256-82-1	b	17.35	146	162	7.97
Vinclozolin	50471-44-8	b	17.44	212	285	13.1
2,4,4'-Trichlorobiphenyl	7012-37-5	g	17.48	256	186	13.7
Parathion methyl	298-00-0	c	17.51	109	263	12.8
Simetryn	1014-70-6	d	17.57	213	170	9.16
Alachlor	15972-60-8	b	17.58	160	188	5.79
Ametryn	834-12-8	b	17.66	227	212	18.0
Prometryne	7287-19-6	d	17.71	241	184	10.8
Heptachlor	76-44-8	a	17.93	272	100	20.1
Terbutryn	886-50-0	d	18.05	226	185	8.06
Bromacil	314-40-9	b	18.11	205	207	47.1
Dibutyl phthalate	84-74-2	e	18.18	149	150	70.3
2,2',5,5'-Tetrachlorobiphenyl	35693-99-3	g	18.28	292	220	12.0
Cyanazine	21725-46-2	b	18.46	225	68	27.5
Chlorpyrifos	2921-88-2	c	18.53	199	97	27.7
Metolachlor	51218-45-2	b	18.53	162	238	9.92
Parathion	56-38-2	c	18.68	291	109	7.40
Dacthal	1861-32-1	a	18.71	301	299	3.66
Triadimefon	43121-43-3	b	18.77	57	208	23.2
2,2',3,5'-Tetrachlorobiphenyl	41464-39-5	g	18.78	292	220	11.5
Aldrin	309-00-2	a	18.86	263	66	18.5
Diphenamid	957-51-7	b	19.06	72	167	16.1
MGK264(a)	113-48-4	b	19.12	164	66	25.0

MGK264(b)	113-48-4	b	19.41	164	66	19.6
Chlorfenvinphos	470-90-6	c	19.56	267	323	38.3
Heptachlor epoxide	1024-57-3	a	19.76	353	81	21.7
2,3',4',5-Tetrachlorobiphenyl	32598-11-1	g	19.78	292	220	9.69
Tetrachlorvinphos	22248-79-9	c	20.25	331	109	26.6
trans-Chlordane	5103-71-2	a	20.34	375	373	7.11
Butachlor	23184-66-9	b	20.35	176	160	15.8
Pyrene	129-00-0	f	20.67	202	200	14.7
cis-Chlordane	5103-71-9	a	20.69	375	373	25.1
Endosulfan I	959-98-8	d	20.71	195	241	19.2
Napropamide	15299-99-7	b	20.73	72	128	7.18
trans-Nonachlor	39765-80-5	a	20.78	409	407	5.57
Profenofos	41198-08-7	c	20.94	339	139	55.6
Oxyfluorfen	42874-03-3	b	21.05	252	361	6.28
Tribufos	78-48-8	c	21.08	169	57	8.06
4,4'-DDE	72-55-9	a	21.10	246	318	10.5
2,3,3',4',6-Pentachlorobiphenyl	38380-03-9	g	21.31	326	254	10.0
Dieldrin	309-00-2	a	21.38	79	81	19.4
Nitrofen	1836-75-5	b	21.69	283	202	41.4
2,2',3,4',5'6-Hexachlorobiphenyl	38380-04-0	g	21.89	360	290	9.51
Chlorobenzilate	510-15-6	a	21.89	251	139	15.3
Endrin	72-20-8	a	21.93	263	81	3.92
2,3',4,4',5-Pentachlorobiphenyl	31508-00-6	g	21.99	326	254	8.94
Ethion	563-12-2	c	22.09	231	97	3.09
Endosulfan II	33213-65-9	d	22.13	195	241	18.6
4,4'-DDD	72-54-8	a	22.15	235	237	6.98
2,2',4,4',5,5'-Hexachlorobiphenyl	35065-27-1	g	22.52	360	290	7.41
Norflurazon	27314-13-2	b	22.76	303	145	3.39
Butylbenzylphthalate	85-68-7	e	22.88	149	91	5.31
Endosulfan sulfate	1031-07-8	d	23.10	272	387	15.8
4,4'-DDT	50-29-3	a	23.14	235	237	2.73
Hexazinone	51235-04-2	b	23.16	171	83	13.7
2,2',3,4,4',5'-Hexachlorobiphenyl	35065-28-2	g	23.21	360	290	7.27
di(2-Ethylhexyl)adipate	103-23-1	e	23.31	129	57	10.1
Tebiconazole	107534-96-3	b	23.46	125	250	19.9
Triphenyl phdhate	115-86-6	h	23.50	326	325	NA
Methoxychlor	72-43-5	a	24.47	227	228	1.49
Benzo[a]anthracene	56-55-3	f	24.55	228	226	6.86
Chrysene	218-01-9	f	24.67	228	226	15.6
2,2',3,4,4',5,5'-Heptachlorobiphenyl	35065-29-3	g	24.90	394	324	6.97

di(2-Ethylhexyl)phthalate	117-81-7	e	24.94	149	167	28.1
Fenarimol	60168-88-9	b	26.02	139	107	9.33
cis-Permethrin	61949-76-6	a	26.76	183	163	3.45
trans-Permethrin	61949-77-7	a	26.92	183	163	2.29
Benzo[b]fluorancene	205-99-2	f	28.01	252	126	3.99
Benzo[k]fluorancene	207-08-9	f	28.01	252	126	6.46
Fluridone	59756-60-4	b	28.70	328	329	7.51
Benzo(a)pyrene-D12	63466-71-7	h	28.80	264	132	NA
Benzo[a]pyrene	50-32-8	f	28.87	252	126	25.2
Dibenzo[a,h]anthracene	53-70-3	f	32.10	276	138	1.11
Indeno[1,2,3-c,d]pyrene	193-39-5	f	32.17	278	139	1.38
Benzo[g,h,i]perylene	191-24-2	f	32.94	276	138	1.52

* a-OCPs; b-ONPs; c-OPPs; d-OSPs, e-Phthalates and others; f-PAHs; g-PCBs; and h-surrogates

Table S2. Comparisons of LODs, accuracy, and RSDs of previous studies using SPME and SBSE coupled with GC-QMS.

References	Methods	Analytes	Matrices	LODs (ng/mL)	Accuracy	RSDs
[1]	SPME	OPPs	Natural waters	0.02-0.04	80.5-124.5%	8-17%
[2]	SPME	Endocrine disruptors	Environmental waters	0.01-0.1	93.0-108.8%	3.1-11.3%
[3]	SPME	Carbamate pesticides	Drinking and surface water	0.6–19	NA	10-17%
[4]	SPME	Triazines	Tap water	14-95	85-99.8%	4.87-10.8%
[5]	SPME	Triazines	Tap water	20-88	96.3-99.6%	6.5-11.6%
[6]	SPME	Semivolatile pesticides	Surface and ground water	0.02-0.3	70.2-108.5%	3.8-14.3%
[7]	SPME	OCPs	Surface water	0.03-800	NA	8-25%
[8]	SPME	Semivolatile pesticides	Mangoes	1.00-3.33	52.85-110.96%	3.15-15.62%
[9]	SBSE	Semivolatile pesticides	River water	0.001-0.1	58.5–133.3%	1.4-30.4%
[10]	SBSE	Semivolatile pesticides	Spinach	0.63-24	NA	NA
[11]	SBSE	PAHs and PCBs	Seawater and interstitial marine water	0.1-50.0	43-123%	0.6-26.6%
[12]	SBSE	Odor compounds	Natural water	0.011–0.071	NA	3.2-18%
[13]	SBSE	Semivolatile pesticides	Baby food	0.5-2	43-100%	4.6-8.8%
[14]	SBSE	Triazole pesticides	Tap water and wastewater	0.53-24	71-110%	2.9-5.2%
[15]	SBSE	OCPs	Purified water	0.02-1.59	88.1-100.0%	2.1-14.8%

Table S3. Recovery of individual analytes in FSPs with sleeves 1, 4, 7, and 10 days after FEVE, relative to the recovery of these analytes analyzed immediately after FEVE (n=3).

	Category*	Day1	Day4	Day7	Day10
HCCPD	a	75.81%	92.15%	92.20%	83.47%
Chlorneb	a	100.65%	91.32%	87.69%	87.33%
a-HCH	a	97.46%	91.41%	89.13%	85.75%
Hexachlorobenzene	a	97.21%	91.94%	91.62%	86.73%
b-HCH	a	97.96%	90.90%	89.91%	85.74%
Pentachlorophenol	a	112.51%	101.19%	101.49%	107.02%
d-HCH	a	99.66%	92.36%	88.88%	84.77%
g-HCH	a	95.44%	92.67%	92.45%	83.99%
Heptachlor	a	97.04%	95.07%	92.21%	89.46%
Aldrin	a	94.92%	90.39%	88.46%	83.71%
Dacthal	a	95.76%	89.73%	89.29%	84.34%
Heptachlor epoxide	a	96.57%	91.26%	89.43%	85.66%
trans-Chlordane	a	98.87%	92.47%	90.35%	86.89%
cis-Chlordane	a	97.16%	92.92%	91.21%	86.64%
trans-Nonachlor	a	97.38%	91.46%	90.44%	86.00%
Dieldrin	a	98.38%	94.72%	91.69%	87.24%
4,4'-DDE	a	96.53%	89.86%	86.30%	80.47%
Endrin	a	99.15%	99.25%	91.28%	94.32%
Chlorobenzilate	a	99.78%	97.49%	95.75%	98.13%
4,4'-DDD	a	98.85%	91.35%	92.55%	86.38%
4,4'-DDT	a	94.56%	117.31%	109.17%	102.55%
Methoxychlor	a	91.24%	90.38%	86.61%	89.13%
cis-Permethrin	a	105.66%	91.74%	89.06%	96.50%
trans-Permethrin	a	104.56%	92.07%	88.10%	96.98%
2,6-Dinitrotoluene	b	99.21%	87.54%	86.41%	82.82%
2,4-Dinitrotoluene	b	99.10%	105.02%	128.24%	118.52%
DEET	b	100.32%	86.30%	80.19%	78.19%
Propachlor	b	107.42%	96.15%	93.22%	88.30%
Chlorpropham	b	102.65%	100.64%	100.61%	100.88%
Trifluralin	b	96.66%	114.62%	114.48%	115.78%
Atraton	b	98.41%	86.13%	77.36%	81.35%
Simazine	b	98.96%	89.92%	84.15%	84.94%
Prometon	b	96.51%	86.59%	76.84%	81.11%
Atrazine	b	97.12%	88.41%	81.51%	80.42%

Propazine	b	97.64%	88.48%	79.59%	79.82%
Pronamide	b	96.71%	87.55%	84.33%	81.68%
Terbacil	b	117.84%	100.93%	93.13%	131.69%
Chlorothalonil	b	99.45%	96.27%	108.26%	99.60%
Acetochlor	b	117.90%	109.66%	117.95%	121.88%
Vinclozolin	b	104.07%	93.28%	87.71%	89.32%
Alachlor	b	102.97%	93.15%	87.37%	86.92%
Ametryn	b	95.87%	88.19%	80.79%	80.43%
Bromacil	b	100.11%	86.48%	82.88%	87.67%
Metolachlor	b	97.63%	90.73%	81.45%	84.12%
Cyanazine	b	103.38%	92.38%	86.57%	94.69%
Triadimefon	b	98.17%	108.21%	112.20%	100.72%
MGK264(a)	b	99.00%	89.86%	87.17%	84.94%
Diphenamid	b	97.16%	89.38%	82.63%	80.46%
MGK264(b)	b	94.04%	92.28%	87.25%	88.13%
Butachlor	b	106.54%	103.72%	100.23%	101.22%
Napropamide	b	97.18%	90.88%	86.59%	86.05%
Oxyfluorfen	b	91.44%	100.92%	128.55%	111.89%
Nitrofen	b	94.84%	98.12%	124.03%	111.47%
Norflurazon	b	104.68%	94.67%	90.29%	102.90%
Hexazinone	b	103.58%	87.96%	85.48%	84.09%
Tebiconazole	b	100.69%	88.68%	84.19%	89.02%
Fenarimol	b	98.25%	86.91%	83.57%	87.10%
Fluridone	b	114.08%	100.90%	111.81%	117.48%
DIMP	c	92.03%	74.10%	58.76%	78.28%
Dichlorvos	c	93.97%	94.99%	82.72%	83.08%
Mevinphos	c	119.10%	103.99%	87.71%	95.27%
Vernolate	c	98.62%	90.53%	88.74%	84.57%
Ethoprop	c	106.65%	97.80%	92.73%	105.40%
Phorate	c	85.28%	79.33%	78.16%	78.87%
Phosphamidon	c	95.54%	72.67%	75.73%	75.47%
Parathion methyl	c	111.54%	107.62%	114.61%	108.68%
Chlorpyrifos	c	96.52%	94.14%	96.66%	89.22%
Parathion	c	92.71%	94.00%	108.77%	100.13%
Chlorfenvinphos	c	113.45%	105.93%	100.32%	100.11%
Tetrachlorvinphos	c	114.28%	126.21%	86.72%	84.30%
Profenofos	c	118.02%	115.16%	102.52%	102.13%
Tribufos	c	109.47%	103.77%	100.53%	103.87%

Ethion	c	98.86%	104.23%	112.41%	115.66%
EPTC	d	98.43%	89.01%	86.54%	81.44%
Butylate	d	98.91%	91.75%	89.79%	85.45%
Etridiazole	d	90.04%	94.11%	89.78%	84.57%
Pebulate	d	99.27%	90.42%	89.17%	84.24%
Tebuthiuron	d	104.72%	95.74%	94.75%	92.22%
Molinate	d	97.58%	87.37%	85.59%	81.08%
Cycloate	d	100.36%	90.98%	88.47%	83.56%
Dimethipin	d	81.54%	103.68%	120.10%	89.08%
Disulfoton	d	96.26%	87.81%	86.72%	82.04%
Simetryn	d	95.98%	88.81%	81.72%	82.34%
Prometryne	d	96.99%	89.24%	80.50%	81.24%
Terbutryn	d	96.71%	90.37%	82.53%	84.36%
Endosulfan I	d	101.43%	98.81%	96.59%	99.07%
Endosulfan II	d	108.08%	101.08%	116.43%	118.81%
Endosulfan sulfate	d	127.81%	119.52%	134.89%	140.03%
Isophorone	e	100.18%	97.76%	87.86%	89.22%
Dimethylphthalate	e	98.46%	86.23%	82.93%	79.91%
BHT	e	94.90%	89.65%	88.76%	85.37%
Diethylphthalate	e	100.19%	98.45%	99.78%	93.72%
Dibutyl phthalate	e	98.30%	90.57%	90.18%	87.39%
Butylbenzylphthalate	e	104.12%	97.52%	97.16%	107.46%
di(2-Ethylhexyl)adipate	e	105.05%	92.77%	91.33%	98.88%
di(2-Ethylhexyl)phthalate	e	103.58%	90.70%	85.08%	91.52%
Acenaphthylene	f	98.00%	90.09%	89.41%	84.41%
Fluorene	f	97.65%	91.73%	92.90%	87.15%
Phenanthrene	f	98.93%	92.06%	91.68%	87.16%
Anthracene	f	99.08%	91.33%	91.64%	87.87%
Pyrene	f	97.97%	92.96%	93.02%	88.93%
Chrysene	f	99.43%	94.51%	93.91%	95.08%
Benzo[a]anthracene	f	93.30%	90.55%	90.86%	85.50%
Benzo[b]fluorancene	f	102.38%	99.80%	99.46%	96.49%
Benzo[k]fluorancene	f	100.55%	94.06%	94.00%	88.08%
Benzo[a]pyrene	f	94.62%	90.46%	89.26%	88.07%
Dibenzo[a,h]anthracene	f	95.18%	92.52%	87.97%	90.22%
Indeno[1,2,3-c,d]pyrene	f	98.78%	91.23%	97.73%	91.75%
Benzo[g,h,i]perylene	f	95.89%	85.92%	81.70%	82.02%
2-Chlorobiphenyl	g	98.32%	93.72%	93.67%	88.05%

4-Chlorobiphenyl	g	98.22%	93.37%	93.55%	88.43%
2,4'-Dichlorobiphenyl	g	97.41%	93.35%	93.51%	88.81%
2,2',5-Trichlorobiphenyl	g	99.56%	93.54%	94.11%	88.34%
2,4,4'-Trichlorobiphenyl	g	97.73%	93.93%	92.42%	87.57%
2,2',5,5'-Tetrachlorobiphenyl	g	99.28%	92.99%	91.24%	88.59%
2,2',3,5'-Tetrachlorobiphenyl	g	95.50%	91.08%	89.52%	86.10%
2,3',4',5-Tetrachlorobiphenyl	g	99.24%	93.33%	91.41%	86.80%
2,3,3',4',6-Pentachlorobiphenyl	g	95.90%	90.09%	88.24%	82.81%
2,2',3,4',5'6-Hexachlorobiphenyl	g	99.18%	90.52%	86.86%	83.77%
2,3',4,4',5-Pentachlorobiphenyl	g	98.68%	91.40%	88.41%	82.51%
2,2',4,4',5,5'-Hexachlorobiphenyl	g	99.02%	89.60%	89.78%	84.02%
2,2',3,4,4',5'-Hexachlorobiphenyl	g	98.02%	90.06%	87.63%	82.52%
2,2',3,4,4',5,5'-Heptachlorobiphenyl	g	96.61%	90.68%	85.37%	82.52%

* a-OCPs; b-ONPs; c-OPPs; d-OSPs, e-Phthalates and others; f-PAHs; and g-PCBs

Table S4. Measurements of 123 target SVOCs in 10 drinking water and surface water samples (n=3). A, B, C, and D were bottled water samples; E was tap water sample; F, G, and I were Creek water samples; and H and J were lake water samples. ND=Not detected.

	A	B	C	D	E	F	G	H	I	J
DIMP	ND	ND	ND	ND	ND	ND	ND	ND	ND	ND
Isophorone	ND	ND	ND	ND	ND	ND	ND	ND	ND	ND
Dichlorvos	ND	ND	ND	ND	ND	ND	ND	ND	ND	155
HCCPD	160	170	160	ND	ND	ND	ND	ND	ND	ND
EPTC	ND	ND	ND	ND	ND	34.5	ND	ND	ND	55.7
Mevinphos	ND	ND	ND	ND	85.8	ND	81.0	ND	ND	86.3
Butylate	104	ND	ND	102	ND	ND	ND	ND	ND	ND
Vernolate	ND	ND	ND	ND	ND	ND	ND	ND	ND	34.1
Dimethylphthalate	ND	ND	ND	ND	ND	ND	ND	ND	ND	ND
2,6-Dinitrotoluene	135	ND	ND	ND	ND	132	ND	ND	ND	ND
Etridiazole	ND	ND	ND	ND	ND	ND	ND	ND	ND	ND
Pebulate	ND	ND	ND	ND	ND	ND	ND	ND	ND	ND
AceNDphthylene	ND	ND	ND	ND	ND	ND	ND	ND	ND	ND
Chlorneb	ND	ND	ND	ND	ND	ND	39.4	ND	ND	ND
BHT	ND	81.7	ND	ND	ND	ND	ND	ND	ND	ND
2-Chlorobiphenyl	26.9	25.6	ND	ND	ND	ND	ND	ND	ND	ND
Tebuthiuron	34.5	30.8	ND	ND	ND	ND	ND	ND	82.9	ND
2,4-Dinitrotoluene	ND	ND	ND	ND	ND	145	ND	ND	ND	ND
Molinate	9.90	ND	ND	6.10	ND	9.53	ND	ND	ND	ND
DEET	19.6	ND	ND	ND	ND	ND	ND	ND	ND	ND
Diethylphthalate	ND	ND	ND	ND	ND	ND	ND	ND	ND	ND
4-Chlorobiphenyl	ND	ND	49.9	ND	ND	56.4	ND	ND	ND	ND
Propachlor	ND	ND	ND	ND	ND	ND	ND	ND	ND	ND
Fluorene	ND	ND	ND	ND	ND	ND	ND	ND	ND	ND
Ethoprop	ND	ND	ND	ND	ND	ND	ND	ND	ND	ND
Cycloate	ND	ND	ND	ND	ND	ND	ND	ND	ND	ND
Chlorpropham	ND	ND	ND	50.0	ND	ND	ND	ND	ND	ND
Trifluralin	ND	ND	ND	ND	ND	ND	ND	ND	ND	ND
Phorate	76.0	ND	ND	ND	ND	ND	ND	ND	ND	ND

a-HCH	ND	ND	ND	ND	ND	ND	ND	ND	ND	ND
2,4'-Dichlorobiphenyl	ND	ND	ND	ND	ND	ND	ND	ND	ND	ND
Atraton	ND	ND	ND	ND	ND	ND	ND	ND	ND	ND
Hexachlorobenzene	ND	ND	ND	ND	ND	75.2	ND	ND	ND	ND
Prometon	ND	ND	ND	ND	ND	ND	ND	ND	131	ND
Simazine	ND	ND	ND	ND	ND	ND	ND	ND	ND	ND
Dimethipin	ND	ND	ND	ND	ND	ND	ND	ND	ND	ND
Atrazine	ND	ND	ND	119	ND	ND	ND	ND	119	ND
Propazine	ND	ND	ND	115	ND	ND	ND	ND	103	ND
b-HCH	ND	11.7	ND	25.2	ND	ND	ND	ND	ND	ND
Pentachlorophenol	ND	ND	ND	ND	ND	ND	ND	ND	ND	ND
d-HCH	ND	ND	ND	ND	50.6	ND	ND	ND	44.7	ND
Pronamide	ND	ND	ND	ND	ND	72.2	ND	ND	ND	ND
Chlorothalonil	ND	ND	ND	ND	ND	ND	ND	ND	ND	ND
2,2',5-Trichlorobiphenyl	ND	ND	ND	9.35	ND	10.5	ND	ND	ND	ND
Terbacil	ND	ND	ND	ND	ND	113	51.7	ND	ND	ND
Disulfoton	ND	ND	ND	ND	ND	ND	ND	ND	ND	ND
Phenanthrene	ND	ND	ND	ND	ND	ND	ND	ND	ND	ND
g-HCH	ND	ND	ND	72.0	ND	49.2	ND	ND	ND	56.9
Anthracene	22.9	ND	ND	34.2	ND	ND	ND	ND	ND	ND
Phosphamidon	ND	ND	ND	ND	ND	ND	ND	ND	ND	ND
Acetochlor	ND	ND	ND	94.0	ND	ND	ND	ND	ND	ND
Vinclozolin	ND	ND	ND	ND	ND	ND	244	ND	ND	ND
2,4,4'-Trichlorobiphenyl	ND	ND	ND	108	ND	103	ND	ND	102	ND
Simetryn	ND	ND	ND	ND	ND	172	ND	ND	204	ND
Alachlor	ND	ND	ND	97.6	ND	75.8	ND	ND	128	ND
Ametryn	ND	ND	ND	ND	ND	ND	167	ND	194	ND
Parathion methyl	ND	ND	ND	ND	ND	150	237	ND	ND	189
Prometryne	ND	ND	ND	ND	ND	ND	ND	ND	ND	ND
Heptachlor	ND	ND	ND	ND	ND	ND	ND	ND	ND	ND
Bromacil	ND	ND	ND	ND	ND	276	6986	ND	266	ND
Terbutryn	ND	ND	ND	ND	ND	ND	ND	ND	202	ND
Dibutyl phthalate	ND	ND	899	ND	ND	604	ND	ND	ND	ND
2,2',5,5'-Tetrachlorobiphenyl	ND	ND	ND	ND	ND	ND	ND	ND	ND	ND

Cyanazine	ND	ND	ND	ND	ND	ND	ND	206	340	ND
Chlorpyrifos	ND	ND	82.4	ND	80.5	ND	205	ND	113	ND
Metolachlor	ND	58.7	ND	60.0	ND	53.1	62.4	ND	90.9	ND
Dacthal	ND	ND	ND	160	ND	ND	ND	ND	ND	ND
Triadimefon	ND	ND	ND	ND	ND	196	ND	ND	432	ND
2,2',3,5'-Tetrachlorobiphenyl	ND	ND	ND	88.4	ND	86.2	ND	ND	69.0	ND
Parathion	ND	ND	ND	ND	ND	ND	ND	ND	ND	ND
Aldrin	ND	ND	116.0	ND	ND	126	ND	ND	127	ND
Diphenamid	ND	ND	ND	ND	ND	ND	ND	ND	ND	ND
MGK264(a)	ND	ND	ND	ND	ND	ND	201	ND	ND	ND
MGK264(b)	ND	ND	ND	ND	ND	ND	301	ND	ND	ND
Chlorfenvinphos	ND	ND	ND	ND	ND	ND	ND	ND	ND	ND
Heptachlor epoxide	ND	60.7	ND	87.5	ND	ND	ND	50.1	85.9	ND
2,3',4',5-Tetrachlorobiphenyl	ND	ND	ND	89.7	ND	84.0	ND	ND	74.4	69.9
Tetrachlorvinphos	ND	ND	ND	ND	ND	ND	ND	ND	ND	ND
trans-Chlordane	ND	ND	ND	126	ND	102	ND	ND	ND	ND
Butachlor	87.4	ND	ND	ND	89.9	ND	92.6	ND	112	ND
cis-Chlordane	ND	ND	ND	ND	ND	ND	ND	ND	ND	ND
Pyrene	ND	ND	ND	ND	ND	ND	ND	ND	ND	ND
Profenofos	ND	ND	ND	ND	ND	ND	ND	ND	ND	ND
Endosulfan I	ND	ND	ND	ND	ND	ND	ND	ND	ND	ND
NDpropamide	ND	ND	ND	ND	ND	ND	ND	ND	ND	ND
trans-Nonachlor	ND	ND	ND	173	ND	ND	ND	ND	ND	ND
Tribufos	ND	ND	ND	ND	ND	225	ND	ND	255	ND
4,4'-DDE	ND	ND	ND	ND	ND	59.0	ND	ND	80.0	ND
2,3,3',4',6-Pentachlorobiphenyl	ND	ND	ND	ND	ND	ND	ND	ND	136	ND
Dieldrin	ND	ND	ND	ND	ND	ND	ND	ND	ND	ND
Nitrofen	ND	ND	314	ND	ND	ND	411	ND	ND	ND
Oxyfluorfen	ND	ND	ND	ND	ND	233	ND	ND	ND	ND
2,2',3,4',5'6-Hexachlorobiphenyl	ND	54.7	ND	ND	ND	66.6	ND	ND	78.9	ND
Chlorobenzilate	ND	ND	87.6	ND	ND	161	ND	ND	151	ND
Endrin	ND	ND	ND	159	ND	206	ND	ND	ND	146
2,3',4,4',5-Pentachlorobiphenyl	ND	ND	ND	ND	ND	ND	ND	ND	118	ND
Ethion	ND	ND	ND	ND	ND	ND	ND	ND	162	ND

Endosulfan II	ND	ND	ND	ND	ND	ND	ND	ND	ND	ND
4,4'-DDD	ND	ND	ND	ND	ND	35.6	ND	ND	50.1	ND
2,2',4,4',5,5'-Hexachlorobiphenyl	ND	ND	ND	141	ND	146	ND	ND	169	ND
Norflurazon	ND	ND	ND	180	ND	ND	ND	ND	265	ND
Butylbenzylphthalate	ND	ND	ND	ND	ND	113	ND	ND	ND	106
Endosulfan sulfate	ND	ND	ND	ND	ND	ND	ND	ND	ND	ND
4,4'-DDT	ND	ND	ND	ND	ND	141	ND	ND	157	ND
Hexazinone	ND	ND	ND	ND	ND	ND	ND	106	ND	140
2,2',3,4,4',5'-Hexachlorobiphenyl	ND	ND	ND	ND	ND	120	ND	ND	148	ND
di(2-Ethylhexyl)adipate	ND	ND	ND	ND	ND	ND	ND	ND	ND	ND
Tebiconazole	ND	ND	ND	ND	ND	ND	ND	ND	208	ND
Methoxychlor	ND	ND	ND	ND	ND	ND	193	ND	202	ND
Benzo[a]anthracene	ND	ND	ND	ND	ND	113	ND	ND	116	ND
Chrysene	ND	ND	ND	104	ND	104	ND	ND	114	ND
2,2',3,4,4',5,5'-Heptachlorobiphenyl	ND	ND	ND	ND	ND	154	ND	ND	212	ND
di(2-Ethylhexyl)phthalate	ND	ND	ND	ND	ND	ND	ND	ND	ND	ND
Fenarimol	ND	ND	ND	ND	ND	ND	ND	ND	ND	ND
cis-Permethrin	ND	ND	ND	ND	ND	ND	ND	ND	ND	ND
trans-Permethrin	ND	ND	ND	ND	ND	ND	ND	ND	ND	ND
Benzo[b]fluorancene	ND	ND	ND	ND	ND	ND	ND	ND	ND	ND
Benzo[k]fluorancene	ND	ND	ND	ND	ND	ND	ND	ND	ND	ND
Fluridone	ND	ND	ND	ND	ND	ND	ND	ND	ND	ND
Benzo[a]pyrene	ND	ND	ND	ND	ND	ND	ND	ND	ND	ND
Dibenzo[a,h]anthracene	ND	ND	ND	ND	ND	ND	ND	ND	ND	ND
Indeno[1,2,3-c,d]pyrene	ND	ND	ND	ND	ND	ND	ND	ND	ND	ND
Benzo[g,h,i]perylene	ND	ND	ND	ND	ND	ND	ND	ND	ND	ND

References:

(1) Lambropoulou, D. A.; Albanis, T. A. Optimization of headspace solid-phase microextraction conditions for the determination of organophosphorus insecticides in natural waters. *J. Chromatogr. A* **2001**, *922* (1-2), 243-255.
(2) Helaleh, M. I.; Fujii, S.; Korenaga, T. Column silylation method for determining endocrine disruptors from environmental water samples by solid phase micro-extraction. *Talanta* **2001**, *54* (6), 1039-1047.
(3) Carabias-Martínez, R.; García-Hermida, C.; Rodríguez-Gonzalo, E.; Ruano-Miguel, L. Behaviour of carbamate pesticides in gas chromatography and their determination with solid-phase extraction and solid-phase microextraction as preconcentration steps. *J. Sep. Sci.* **2005**, *28* (16), 2130-2138.
(4) Djozan, D.; Mahkam, M.; Ebrahimi, B. Preparation and binding study of solid-phase microextraction fiber on the basis of ametryn-imprinted polymer: application to the selective extraction of persistent triazine herbicides in tap water, rice, maize and onion. *J. Chromatogr. A* **2009**, *1216* (12), 2211-2219.
(5) Djozan, D.; Ebrahimi, B. Preparation of new solid phase micro extraction fiber on the basis of atrazine-molecular imprinted polymer: application for GC and GC/MS screening of triazine herbicides in water, rice and onion. *Anal. Chim. Acta* **2008**, *616* (2), 152-159.
(6) Menezes Filho, A.; dos Santos, F. N.; Pereira, P. A. d. P. Development, validation and application of a method based on DI-SPME and GC–MS for determination of pesticides of different chemical groups in surface and groundwater samples. *Microchem. J.* **2010**, *96* (1), 139-145.
(7) Magdic, S.; Pawliszyn, J. B. Analysis of organochlorine pesticides using solid-phase microextraction. *J. Chromatogr. A* **1996**, *723* (1), 111-122.
(8) Menezes Filho, A.; dos Santos, F. N.; de Paula Pereira, P. A. Development, validation and application of a methodology based on solid-phase micro extraction followed by gas chromatography coupled to mass spectrometry (SPME/GC–MS) for the determination of pesticide residues in mangoes. *Talanta* **2010**, *81* (1-2), 346-354.
(9) Nakamura, S.; Daishima, S. Simultaneous determination of 64 pesticides in river water by stir bar sorptive extraction and thermal desorption-gas chromatography-mass spectrometry. *Anal. Bioanal. Chem.* **2005**, *382* (1), 99-107.
(10) Ochiai, N.; Sasamoto, K.; Kanda, H.; Yamagami, T.; David, F.; Tienpont, B.; Sandra, P. Optimization of a multi-residue screening method for the determination of 85 pesticides in selected food matrices by stir bar sorptive extraction and thermal desorption GC-MS. *J. Sep. Sci.* **2005**, *28* (9-10), 1083-1092.
(11) Pérez-Carrera, E.; León, V. M. L.; Parra, A. G.; González-Mazo, E. Simultaneous determination of pesticides, polycyclic aromatic hydrocarbons and polychlorinated biphenyls in seawater and interstitial marine water samples, using stir bar sorptive extraction–thermal desorption–gas chromatography–mass spectrometry. *J. Chromatogr. A* **2007**, *1170* (1-2), 82-90.
(12) Ochiai, N.; Sasamoto, K.; Ieda, T.; David, F.; Sandra, P. Multi-stir bar sorptive extraction for analysis of odor compounds in aqueous samples. *J. Chromatogr. A* **2013**, *1315*, 70-79.
(13) Sandra, P.; Tienpont, B.; David, F. Multi-residue screening of pesticides in vegetables, fruits and baby food by stir bar sorptive extraction–thermal desorption–capillary gas chromatography–mass spectrometry. *J. Chromatogr. A* **2003**, *1000* (1-2), 299-309.

(14) Farajzadeh, M. A.; Djozan, D.; Nouri, N.; Bamorowat, M.; Shalamzari, M. S. Coupling stir bar sorptive extraction-dispersive liquid–liquid microextraction for preconcentration of triazole pesticides from aqueous samples followed by GC-FID and GC-MS determinations. *J. Sep. Sci.* **2010**, *33* (12), 1816-1828.
(15) Grossi, P.; Olivares, I. R.; de Freitas, D. R.; Lancas, F. M. A novel HS-SBSE system coupled with gas chromatography and mass spectrometry for the analysis of organochlorine pesticides in water samples. *J. Sep. Sci.* **2008**, *31* (20), 3630-3637.
